# LeishIF3d is a non-canonical cap-binding protein in *Leishmania*

**DOI:** 10.3389/fmolb.2023.1191934

**Published:** 2023-05-30

**Authors:** Priyanka Bose, Nofar Baron, Durgeshwar Pullaiahgari, Anat Ben-Zvi, Michal Shapira

**Affiliations:** Department of Life Sciences, Ben-Gurion University of the Negev, Beer Sheva, Israel

**Keywords:** LeishIF3d, translation, cap-binding activity, *Leishmania*, trypanosomatids

## Abstract

Translation of most cellular mRNAs in eukaryotes proceeds through a cap-dependent pathway, whereby the cap-binding complex, eIF4F, anchors the pre-initiation complex at the 5′ end of mRNAs driving translation initiation. The genome of *Leishmania* encodes a large repertoire of cap-binding complexes that fulfill a variety of functions possibly involved in survival along the life cycle. However, most of these complexes function in the promastigote life form that resides in the sand fly vector and decrease their activity in amastigotes, the mammalian life form. Here we examined the possibility that LeishIF3d drives translation in *Leishmania* using alternative pathways. We describe a non-canonical cap-binding activity of LeishIF3d and examine its potential role in driving translation. LeishIF3d is required for translation, as reducing its expression by a hemizygous deletion reduces the translation activity of the LeishIF3d(+/−) mutant cells. Proteomic analysis of the mutant cells highlights the reduced expression of flagellar and cytoskeletal proteins, as reflected in the morphological changes observed in the mutant cells. Targeted mutations in two predicted alpha helices diminish the cap-binding activity of LeishIF3d. Overall, LeishIF3d could serve as a driving force for alternative translation pathways, although it does not seem to offer an alternative pathway for translation in amastigotes.

## Introduction

Translation initiation is a rate-limiting step during protein synthesis, affecting the expression of all cellular proteins. Translation of most mRNAs initiates via a cap-dependent pathway, in which a cap-binding complex assembles on the 5′cap structure while recruiting the small ribosomal subunit. The cap-binding complex comprises of a cap-binding protein, eIF4E, an RNA helicase, eIF4A, and a scaffold protein, eIF4G, that serves as a hub for the complex, as it binds the eIF4E, eIG4A, the PolyA binding protein at the 3′end of the mRNA, and eIF3, accompanying the 40S ribosomal subunit (Gingras et al., 1999).

The mammalian eIF3 complex is a large complex comprised of 13 subunits, eight of which (a, c, e, f, h, k, l, and m) serve as a core for the eIF3 complex. These contain the conserved PCI or MPN domains, forming a PCI/MPN octamer (Hinnebusch, 2006; Sun et al., 2011; Cate, 2017; Moitra et al., 2019). These eight subunits share a high degree of conservation with components of the proteasome lid and the COP9 signalosome, known as the PCI complexes (Wee et al., 2002). The remaining subunits eIF3b, g, i and C-terminal region of octameric eIF3a form Yeast-Like-Core (YLC); the remaining peripheral eIF3d subunit attaches to eIF3 via eIF3e (Querol-Audi et al., 2013; Wagner et al., 2016). RNAi studies showed that subunits “a” and “b” serve as the nucleation core for assembling the other subunits. eIF3b was shown to be a key component for the assembly process, whereas the knockdown of eIF3d led to severe proliferation defects, but its absence hardly affected the eIF3 integrity (Wagner et al., 2016).

### Role of eIF3 in translation

The eIF3 complex accompanies the 40S ribosomal subunit and is, therefore, a key player in the assembly of the PIC of translation. The canonical PIC assembles over the mRNA 5′ cap to further scan the 5′ UTR until it reaches the first AUG codon, where the 60S subunit joins, and translation starts. In addition to the canonical scanning process, eIF3 promotes cap-independent translation through binding to the Internal Ribosome Entry Sites (IRES). The complex that binds the IRES recruits the 40S subunit to a site adjacent to the AUG start codon without involving the cap-binding complex (Thompson, 2012; Walters et al., 2020).

It was previously reported that once the ribosome assembles on the AUG start site entering the elongation process, most translation initiation factors disassemble from the complex. However, despite being a key factor in the initiation process, the eIF3 complex continues to be associated with the ribosomes during elongation, although its role during this stage is not fully clear (Hinnebusch, 2006; Valasek et al., 2017; Lin et al., 2020; Wolf et al., 2020).

### Functional examination of eIF3d in human cells

It has been shown that cap-dependent translation is inhibited in response to stress conditions or nutrient deprivation that limits the availability of glucose (Lamper et al., 2020). However, despite this inhibition, certain transcripts continued to be translated under such conditions through alternative pathways. *In vitro* cross-linking experiments revealed that eIF3d bound the 5′ UTR of the *c-Jun* transcription factor, Raptor, an essential component of the mTOR complex 1 and La-related protein 1 (Larp1), a protein that blocks translation of 5′terminal oligopyrimidine motif-containing RNAs during mTOR inhibition (Lamper et al., 2020). Further binding experiments revealed that the eIF3d subunit could bind directly to the 5′ cap structure, replacing the inactivated eIF4F complex. Structural studies of eIF3d revealed that it contained an “RNA gate” motif that is usually known to block access to the 5′ cap-binding pocket and that this block could be surpassed by changes in the RNA structure in the 5′ UTR of *c-Jun* mRNA, allowing eIF3d to bind its 5′ cap-structure (Lee et al., 2016). Sequence alignment of eIF3d from different species revealed the presence of a structural pocket that resembles the canonical cap-binding pocket in eIF4E, which could drive the translation of specific mRNAs involving transcripts that contain complex RNA structures in their 5′ UTRs. Structural analysis showed that two conserved alpha-helices are located near the cap-binding pocket. Mutational analysis indicated that these helices are required for the cap-binding activity of eIF3d (Lee et al., 2016).

### Cap-binding complexes in *Leishmania* and the search for alternative pathways

*Leishmanias* differentiate from flagellated promastigotes found as extracellular cells in the alimentary canal of sand fly vectors to obligatory intracellular aflagellated amastigotes that reside within phagolysosomal vacuoles of mammalian macrophages (Sacks and Perkins, 1984). The parasites are transmitted by female sandflies to their mammalian hosts during their blood meal, exposing them to alternating environmental conditions, including temperature and pH shifts (Zilberstein and Shapira, 1994). Stage differentiation can be mimicked *in vitro* under axenic conditions by temperature elevation (25−>37°C) and pH acidification (8−>5.5), which resemble the conditions in the mammalian host (Zilberstein and Shapira, 1994).

Despite being a unicellular diploid organism with no known sexual reproduction cycle, the *Leishmania* genome encodes a relatively high number of cap-binding complexes based on six eIF4E orthologs (LeishIF4Es 1-6) and five eIF4G candidates (LeishIF4Gs 1–5) (Yoffe et al., 2004; Dhalia et al., 2005; Yoffe et al., 2006; Yoffe et al., 2009; Zinoviev et al., 2011; Moura et al., 2015; Freire et al., 2017; Shrivastava et al., 2019a; Shrivastava et al., 2021; Tupperwar et al., 2021). Affinity chromatography studies indicated that the cap-binding activity of the different LeishIF4Es is inactivated under conditions that induce axenic differentiation. An exception is LeishIF4E1, which binds m^7^GTP efficiently in promastigotes and even in axenic amastigotes, after exposure to elevated temperatures and reduced pH conditions (Zinoviev et al., 2011). Therefore, the possibility that alternative translation pathways prevail in these fascinating organisms convinced us to investigate the possibility that LeishIF3 could be involved in driving unusual translation processes.

### LeishIF3 subunits in *Leishmania* and their unusual interactions with translation factors

The LeishIF3 complex includes 12 subunits, “a-l,” missing only the “m” subunit. Subunits “a” and “c” interact with each other, as in the mammalian complex. However, unique and non-conserved interactions were reported between LeishIF3a and LeishIF4E1 (Meleppattu et al., 2015). Since LeishIF4E1 does not pair with any LeishIF4G candidates, its interaction with the LeishIF3 “a” subunit could explain how the LeishIF3 complex is recruited to the 5′ cap-anchored PIC. Here we examine the cap-binding activity of the *Leishmania* LeishIF3d subunit, providing potential pathways for its function during translation.

## Materials and methods

### Organisms and cell culture

*Leishmania mexicana* M379 promastigotes were routinely cultured in Medium 199 [M199, (pH 7.4)] supplemented with 10% fetal calf serum (FCS, Biological Industries Beit Ha’emek, Israel), 5 μg/mL hemin (Sigma, Israel), 0.1 mM adenine (Sigma, Israel), 40 mM HEPES, 4 mM L-glutamine, 100 U/mL penicillin and 100 μg/mL streptomycin (Biological Industries, Beit Ha’emek, Israel) at 25°C. To differentiate promastigote cells into axenic amastigotes, late log phase promastigotes (3.5*10^7^ cells/mL) were washed twice with phosphate buffered saline (PBS) and resuspended in M199, containing 25% FCS, 5 μg/mL hemin, 0.1 mM adenine, 40 mM HEPES, (adjusted to pH 5.5 using 0.5 M succinic acid), 4 mM L-glutamine, 100 U/mL penicillin and 100 μg/mL streptomycin. Cells were grown at 33°C for 4 days.

### Cloning and transfection

The LeishIF3d open reading frame (ORF, 1596 bp) was amplified from *L. mexicana* genomic DNA using gene-specific primers (Supplementary Table S1). The ORF was cloned into the pX-derived transfection cassette, pX-H-target ORF-H-SBP between the BamHI/XbaI sites. In this transfection cassette H represents the intergenic region of the HSP83 genomic locus (Zinoviev et al., 2011) of *Leishmania*, and a 6 kDa streptavidin-binding peptide, SBP, tag is fused to the C-terminus of the target gene ORF.

To introduce mutations that disrupted the alpha helix structures in LeishIF3d that were previously shown to affect m^7^GTP binding (Lee et al., 2016). The pX-H-LIF3d ORF-H-SBP plasmid was used as a template for one-step mutagenesis PCR using 2 different sets of primers (Supplementary Table S1). This reaction resulted in plasmids pX-H-LeishIF3d L234P-SBP-H and pX-H-LeishIF3d A413P-SBP-H.

Cells were transfected following published protocols (Laban and Wirth, 1989). Mid-log phase *L. mexicana* cells (50 mL) were transfected with 20 µg of each plasmid and selected for resistance to G418 (200 μg/mL). Generation of cells expressing the SBP-tagged LeishIF3e cells was described previously (Meleppattu et al., 2015).

### Generation of transgenic *Leishmania* lines

#### CRISPR-Cas9-mediated deletion of LeishIF3d

Plasmids developed for the CRISPR system in *Leishmania* were obtained from Eva Gluenz (University of Oxford, United Kingdom) (Beneke et al., 2017). The pTB007 plasmid contained the genes encoding the *Streptococcus pyogenes* CRISPR-associated protein 9 endonuclease and the T7 RNA polymerase gene (Cas9/T7), along with the hygromycin resistance gene. pTB007 was transfected into *L. mexicana* promastigotes and transgenic stably transfected cells were selected for hygromycin resistance (200 g/mL).

#### Generation of the LeishIF3d deletion mutant by CRISPR-Cas9

To generate LeishIF3d(+/−) mutants, we used two PCR fragments designed to create double-strand breaks (DSBs) upstream and downstream of the LeishIF3d coding region (5′ and 3′ sgRNAs), and a fragment encoding the LeishIF3d repair cassette containing the blasticidin resistance marker. The three PCR products (4 μg of each) were transfected into mid-log phase transgenic cells expressing Cas9 and T7 RNA polymerase, and cells were further selected for resistance to 20 μg/mL blasticidin (Laban and Wirth, 1989). The sgRNA sequences used to delete the LeishIF3d gene were obtained from LeishGEdit.net (Peng and Tarleton, 2015). The sgRNAs contained the highest-scoring 20 nt fragments located within 105 bp upstream or downstream of the target gene. The sequences of the sgRNAs were blasted against the *L. mexicana* genome in TriTrypDB to verify that the sgRNAs were exclusively specific for LeishIF3d (E value = 0.001 and 8e^−5^). We also ran a BLAST analysis with the drug resistance repair cassette that contained the homology sequence to the UTR of LeishIF3d to target the insertion of the selection marker specifically. The sequences included in the repair cassette showed an E value of 5e^−9^ with the endogenous genome, suggesting very high specificity of the repair sequences. The sgRNA target sequences and the homology arms on the repair cassette fully matched the target sequence of LeishIF3d.

#### PCR amplification of sgRNA templates

DNA fragments encoding LeishIF3d specific 5′ and 3′ guide RNAs for cleavage upstream and downstream to the LeishIF3d target gene were generated. All primers are listed in the Supplementary Table S1. The template for this PCR reaction consisted of two fragments: one contained the common sgRNA scaffold fragment; the other included the T7 RNA polymerase promoter (small letters) fused to the gRNA (5′ or 3′) targeting LeishIF3d (capital letters) followed by a short sequence overlapping with the scaffold fragment (small letters, 5′gRNA or 3′gRNA (LmxM.29.3040, Supplementary Table S1). Each of these two fragments (1 μM each) was annealed to the partially overlapping scaffold fragment and further amplified with two primers derived from the T7 promoter (G00F) and the common scaffold fragment (G00R, Supplementary Table S1, 2 μM each). The reaction mixture consisted of dNTPs (0.2 mM), HiFi Polymerase (1 unit, Phusion, NEB) in GC buffer with MgCl_2_ (NEB), in a total volume of 50 μL. The PCR conditions included an initial denaturation at 98°C for 2 min, followed by 35 cycles of 98°C for 10 s, annealing at 60°C for 30 s, and extension at 72°C for 15 s. All PCR products were gel-purified and heated at 94°C for 5 min before transfection.

#### PCR amplification of the LeishIF3d replacement fragment

A DNA fragment designed to repair the deleted LeishIF3d target gene was amplified by PCR. The LeishIF3d specific primers were derived from the 5′ and 3′ UTR sequences upstream and downstream to the LeishIF3d gene, based on the LeishGEdit database (http://www.leishgedit.net/Home.html) and the sequences of the antibiotic repair cassette. The primers were Upstream Forward (LmxM.29.3040) and Downstream reverse (LmxM.29.3040, Supplementary Table S1). Capital letters represent the UTR sequences of the LeishIF3d gene, and small letters represent the region in the pTNeo plasmid that flanks the antibiotic resistance gene. The PCR for generating the fragment used for repair of the DSBs on both sides of the target gene was performed using the pTNeo plasmid as a template. The resulting fragment promotes the integration of the drug resistance marker by homologous recombination into the target site. The reaction mixture consisted of 2 μM of each primer, dNTPs (0.2 mM), the template pTNeo (30 ng), 3% (v/v) DMSO, HiFi Polymerase (1 U of Phusion, NEB) in GC buffer (with MgCl2 to a final concentration of 1.5 mM) in a total volume of 50 μL. PCR conditions included initial denaturation at 98°C for 4 min followed by 40 cycles of 98°C for 30 s, annealing at 65°C for 30 s, and extension at 72°C for 2 min 15 s. The final extension was performed for 7 min at 72°C. All PCR products were gel purified and heated at 94°C for 5 min before transfection.

#### Diagnostic PCR to confirm the deletion of LeishIF3d

Genomic DNA from the drug-resistant cells was isolated 14 days post-transfection using DNeasy Blood & Tissue Kit (Qiagen) and analyzed for the presence of the LeishIF3d gene using specific primers derived from the UTR of LeishIF3d. The primers used were LeishIF3d forward (5′UTR, P3) and blasticidin Reverse (P2). A parallel reaction was performed to detect the presence of the blasticidin resistance gene with primers derived from its ORF: blasticidin Forward (P1) and blasticidin Reverse (P2). Another PCR was performed with LeishIF3d ORF (P4/P5) to confirm one allele of LeishIF3d is still present. All primers are listed in the Supplementary Table S1. Genomic DNA from Cas9/T7 *L. mexicana* cells was used to detect the presence of the LeishIF3d gene. The reaction mixture consisted of 2 μM of each primer, gDNA (100 ng), dNTPs (0.2 mM), HiFi Polymerase (1 U Phusion, NEB) in GC buffer with MgCl_2_ (NEB) in a total volume of 50 μL. PCR conditions included initial denaturation at 98°C for 4 min followed by 35 cycles of 98°C for 30 s, annealing at 60°C for 30 s, and extension at 72°C for 2 min 15 s. The final extension was done for 7 min at 72°C. PCR products were separated on 1% agarose gels.

### Growth analysis

*L. mexicana* M379 wild-type and Cas9/T7-expressing cells, along with the LeishIF3d(+/−) deletion mutant and the SBP-tagged LeishIF3d, LeishIF3d L234P, LeishIF3d A413P cells, were cultured as promastigotes at 25°C in M199 containing all supplements (see above). Cells were seeded at a concentration of 5*10^5^ cells/mL, and the cells were counted daily for five consecutive days. The curves were obtained from three independent repeats.

### Affinity purification of recombinant LeishIF3d

The open reading frame (ORF) of LeishIF3d was amplified with primers IF3d-BamHI Gibson forward and IF3d HindIII Gibson reverse (Supplementary Table S1) and cloned into the pET52 vector. The protein was expressed in *E. coli* BL21 cells. The bacterial cells were grown to OD_600_ of 0.5–0.7, and expression was induced by adding 0.5 mM IPTG at 37°C for 3 hrs. The cells were harvested and re-suspended in 20 mL lysis buffer (50 mM Tris-HCL pH 8, 200 mM NaCl, 8 mM 2-Mercaptoethanol, 0.01% Triton X-100, 10% Glycerol, a cocktail of protease inhibitors (Sigma, Israel) and 5 μg/mL DNaseI (NEB). The cells were disrupted 3 times in a French Press at 1,500 psi, followed by centrifugation at 45,000 rpm (Beckman 70 Ti rotor) for 45 min at 4°C. The supernatant was further incubated with Ni-NTA beads (Cube Biotech, 5 mL) that were pre-washed two times with Binding Buffer (BB, 50 mM Tris-HCl pH 8, 200 mM NaCl). The supernatant was incubated with the beads for 10 min at 4°C, and the beads were washed with a wash buffer (WB) containing 50 mM Tris-HCl pH 8, 10% Glycerol, 10 mM imidazole, and 500 mM NaCl. Further washes were done with the WB that contained gradually reduced concentrations of NaCl (250 mM and 100 mM). Finally, the beads were eluted with a single column volume (CV) of PBS containing 300 mM imidazole in 20 mM Tris-HCl pH 8, 100 mM NaCl, and 5% Glycerol. The recombinant protein was dialyzed overnight at 4°C against the Binding Buffer. Purified recombinant LIF3d protein was used to commercially generate specific antibodies against LeishIF3d (Adar Biotech, http://www.adarbiotech.com/), that were diluted 1:5,000.

### Affinity purification of transgenic and recombinant LeishIF3d over m^7^GTP-agarose

Wild-type *L. mexicana* cells and transgenic lines expressing the episomal SBP-tagged LeishIF3d, along with its mutant versions of the alpha helices 5 and 11, LeishIF3d L234P, LeishIF3d A413P, accordingly. Control lines expressed LeishIF3e and the luciferase gene (Shrivastava et al., 2019a). Parallel cells were assayed, in which the endogenous LeishIF3d was tagged with a mNeonGreen tag. The cells (∼10^9^) were washed twice with PBS and once with column buffer (CB, 20 mM HEPES, pH 7.4, 2 mM EDTA, 1 mM DTT, and 50 mM NaCl). The cell pellets were re-suspended in 1.2 mL of CB+, which consisted of CB containing a cocktail of protease inhibitors (Sigma, Israel) along with 4 mM iodoacetamide. The mix also contained, 25 mM sodium fluoride and 55 mM β-glycerophosphate (Sigma-Aldrich) as phosphatase inhibitors. *Leishmania* cells were lysed with 1% Triton X-100 in CB + on ice for 5 min. The supernatants were clarified by centrifugation at 20,000 g for 20 min at 4°C. The clarified supernatants were then incubated for 2 h with m^7^GTP-agarose resin (75 µL) (Jena Biosciences, Jena, Germany) pre-equilibrated with CB. The m^7^GTP-agarose resin was then washed with CB twice and once with CB supplemented with 100 µM GTP. Finally, the elution of the cap-binding complexes was carried out with CB + containing 200 µM of free m^7^GTP. The eluted fractions were precipitated with trichloroacetic acid (TCA) at a final concentration of 10% at 4°C overnight, under constant agitation. The proteins were spun down at 20,000 × *g* for 20 min at 4°C, and the pellets were washed with 100% chilled acetone, briefly dried, and resuspended in Laemmli sample buffer. Aliquots derived from the supernatant (1%), the flow-through (1%), wash (50%), and elution (50%) fractions were resolved over 10% or 12% SDS-PAGE gels, and further processed for western analysis performed with specific antibodies. Full blots are provided in the supplemental files (Supplementary Presentation S1).

### Immunoprecipitation of mNeonGreen-tagged LeishIF3d over Myc-agarose beads

Lysates were prepared from transgenic cells in which the endogenous LeishIF3d was tagged with the mNeonGreen tag and from control cells that overexpressed the mNeonGreen tag alone. The cells (∼10^9^) were washed twice with PBS and then lysed in 500 µL of 1% Triton X-100 in 500 µL PRS+(35 mM HEPES, 100 mM KCl, 10 mM MgCl_2_, a cocktail of 2× protease inhibitors, 2 mM iodoacetamide and 1 mM dithiothreitol along with 20 mM NaF, 50 mM β-glycerophosphate) for 5 min in ice. The cell lysates were clarified by centrifugation at 20,000 × *g* for 20 min at 4°C. The clarified supernatants (500 µL each) were then incubated for 2 h with 80 µL of anti-c-Myc-agarose (Pierce) for 2 h. The beads were then washed three times with PRS + containing 0.05% Tween-20. The protein was eluted by adding one-bed volume of non-reducing 2X SDS-PAGE loading buffer (following the commercial instructions). The eluted samples were then subjected to mass spectrometry (MS) analysis.

### Whole cell proteome analysis

To characterize the proteomic differences between the LeishIF3d(+/−) and the Cas9/T7 control cells, we performed MS analysis of total cell lysates. Whole cell lysates from mid-log stage promastigotes of Cas9/T7 and LeishIF3d(+/−) were resuspended in a buffer containing 100 mM Tris HCl pH 7.4, 10 mM DTT, 5% SDS, 2 mM iodoacetamide and a cocktail of protease inhibitors (Sigma, Israel). Cell lysates were precipitated using 10% trichloroacetic acid (TCA), and the pellets were washed with 80% acetone and resolved over 10% SDS-PAGE for further processing. The mass spectrometric analysis was performed by the Smoler Proteomics Center at Technion, Israel. The mass spectrometry proteomics data have been deposited to the ProteomeXchange Consortium via the PRIDE partner repository with the dataset identifier PXD040501 (Perez-Riverol et al., 2022).

### Mass spectrometry analysis

Proteins were reduced using 3 mM DTT (60°C for 30 min), followed by modification with 10 mM iodoacetamide in 100 mM ammonium bicarbonate for 30 min at room temperature. This was followed by overnight digestion in 10 mM ammonium bicarbonate in trypsin (Promega) at 37°C. Trypsin-digested peptides were desalted, dried, resuspended in 0.1% formic acid, and resolved by reverse phase chromatography over a 30 min linear gradient with 5%–35% acetonitrile and 0.1% formic acid in water, a 15 min gradient with 35%–95% acetonitrile and 0.1% formic acid in water and a 15 min gradient at 95% acetonitrile and 0.1% formic acid in water at a flow rate of 0.15 μL/min. MS was performed using a Q-Exactive Plus Mass Spectrometer (Thermo) in a positive mode to conduct a repetitively full MS scan, followed by high energy collision dissociation of the 10 dominant ions selected from the first MS scan. A mass tolerance of 10 ppm for precursor masses and 20 ppm for fragment ions was set.

### Statistical analysis for enriched proteins

Raw MS data were analyzed by the MaxQuant software, version 1.5.2.8 (https://www.maxquant.org/) (Cox and Mann, 2008). The data were searched against the annotated *L. mexicana* proteins from the TriTrypDB (Aslett et al., 2010). Protein identification was set at less than a 1% false discovery rate. The MaxQuant settings selected were a minimum of 1 razor/unique peptide for identification with a minimum peptide length of six amino acids and a maximum of two mis-cleavages. For protein quantification, summed peptide intensities were used. The log2 of LFQ intensities (Krey et al., 2014) were compared between the three biological repeats of each group on the Perseus software platform (Tyanova et al., 2016), using a *t*-test. The enrichment threshold was set to a log2 fold change >1 and q value <0.05. The annotated proteins were categorized manually and by GO annotation performed by TriTrypDB.

### Global translation assay

Global translation was monitored using the non-radioactive SUnSET (Surface SEnsing of Translation) assay. This assay is based on the incorporation of puromycin, an amino-acyl tRNA analog, into the A site of translating ribosomes (Goodman and Hornberger, 2013). Cells were incubated with puromycin (1 μg/mL, Sigma) for 1 h and then washed twice with PBS. Cell pellets were resuspended in 300 μL of PBS, denatured in Laemmli sample buffer, and boiled for 5 min. Cells treated with cycloheximide (100 μg/mL) before adding puromycin served as a negative control. Samples were resolved over 10% SDS-Polyacrylamide gel electrophoresis (SDS-PAGE). The gels were blotted and subjected to western analysis using monoclonal mouse anti-puromycin antibodies (DSHB, 1:1,000) and secondary antibodies of peroxidase-labeled anti-mouse (KPL, 1:10,000).

### Phase contrast microscopy of *Leishmania* promastigotes

Late log-phase cells from different lines were harvested, washed in cold PBS, fixed in 2% paraformaldehyde in PBS, and mounted on glass slides. Phase contrast microscope images were captured at ×100 magnification with a Zeiss Axiovert 200M microscope equipped with an AxioCam HRm CCD camera.

### Sucrose gradients and polysome analysis

*L. mexicana* LeishIF3d(+/−) cells and control promastigotes expressing Cas9/T7 (2*10^8^/gradient) were pelleted at 4,000 rpm for 5 min at 4°C and resuspended in 10 mL of M199 medium containing 100 μg/mL cycloheximide for 5 min. The cells were washed twice in PBS containing 100 μg/mL cycloheximide and once with polysome buffer (15 mM Tris-HCl, pH 7.4, 0.3 M KCl, 5 mM MgCl2, 0.5 mM DTT, 100 μg/mL cycloheximide, and 1 mg/mL heparin). The cell pellet was lysed in polysome buffer with 1% Triton X-100 and incubated on ice for 10 min. The cell lysates were centrifuged at 14,000 rpm at 4°C for 15 min 1 mL of clarified lysate was loaded on 11.5 mL 10%–50% sucrose step gradient prepared in polysome buffer that contained 0.5 mg/mL heparin. The lysates were centrifuged at 35,000 rpm for 160 min at 4°C in a SW40 rotor. Fractions (300 μL) were collected from the top, and the optical density at 260 nm was monitored.

### *L. mexicana* IF3d structural homology modeling

Structural homology of *L. mexicana* IF3d (143–505 aa; LmxM.29.3040) was modeled via the AlphaFold server Superposition of the LeishIF3d predicted structure and the *Nasonia vitripennis (N. vitripennis)* structure of eIF3d (5k4b) was performed using ChimeraX. To validate the alpha fold model an additional model of *L. mexicana* LeishIF3d was generated using the SWISS-MODEL server, based on the *N. vitripennis* eIF3d solved structure (5k4b).

## Results

### Identification of a novel cap-binding protein from *Leishmania*

**FIGURE 1:**
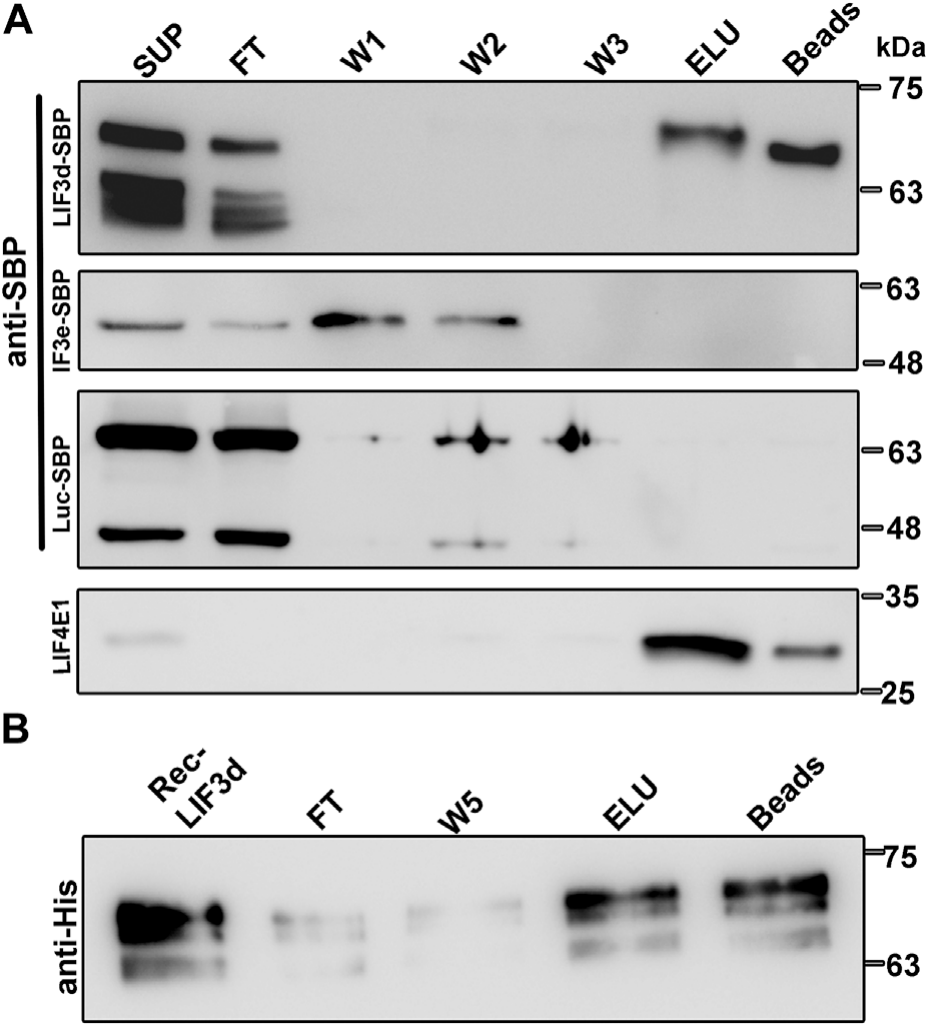
Transgenic and recombinant LeishIF3d bind m^7^GTP. **(A)** Extracts of promastigote cells (10^9^) expressing the episomal SBP-tagged LeishIF3d (LIF3d), LeishIF3e (LIF3e), and Luciferase were incubated with m^7^GTP-agarose beads. The beads were then washed and eluted with free m^7^GTP. Following elution, the beads were boiled in SDS-PAGE sample buffer. Aliquots of the cell extracts (SUP, 1%), flow-through (FT, 1%), the three washes (W1-3, 50%), the eluted fraction (ELU, 50%), and the proteins that remained on the beads (Beads, 50%), were separated over 12% SDS–PAGE. Blots were analyzed using antibodies against the streptavidin binding protein tag (SBP) and antibodies specific for LeishIF4E1 (LIF4E1). **(B)** Recombinant HIS-tagged LeishIF3d was first purified over Ni-NTA column (Rec LIF3d) and then affinity purified over m^7^GTP-agarose. After collecting the FT fraction, the beads were washed (X5) and the bound recombinant protein was eluted with free m^7^GTP (ELU). The protein that remained on the beads was also eluted by boiling in SDS-PAGE sample buffer. Aliquots of the recombinant LIF3d (0.15%), flow-through (FT, 0.15%), the fifth wash (W5, 3%), the eluted fraction (ELU, 3%), and the aliquot that remained on the beads (3%) were resolved over 12% SDS–PAGE and subjected to western analysis using antibodies against the HIS tag.

In attempt to understand how the translation initiation complexes are formed under different physiological conditions and based on the finding in mammalian cells that eIF3d can bind the cap structure (Lee et al., 2016), we examined the cap-binding activity of LeishIF3d. Using extracts of mid-log phase transgenic cell lines expressing SBP-tagged LeishIF3d, control cells expressing SBP-tagged luciferase and LeishIF3e were affinity purified over m^7^GTP-agarose beads. The bound protein complexes were eluted using free m^7^GTP and aliquots from the supernatant, flow through, wash, eluted fractions and beads were subjected to western blot analysis using specific antibodies against the SBP-tag and against LeishIF4E1 (Figure 1A). The results show that LeishIF3d could bind m^7^GTP, as did the positive control of LeishIF4E1, whereas LeishIF3e, another subunit of the LIF3 complex, did not bind m^7^GTP.

To exclude the possibility that LIF3d subunits bind m^7^GTP indirectly through functional cap-binding proteins, we further demonstrated the direct binding of recombinant His-tagged LeishIF3d to m^7^GTP (Supplementary Figure S1). The protein was loaded on m^7^GTP-agarose resin, and the beads were washed and eluted with free m^7^GTP. Aliquots from the different fractions were examined by SDS-PAGE and followed by western blot analysis using anti-His antibodies (Figure 1B). The direct cap-binding activity of LeishIF3d was confirmed.

### LeishIF3d binds m^7^GTP mainly in promastigotes

Since the binding of transgenic SBP-tagged LeishIF3d *in vivo* was performed with cells that were transfected with an episome that can be amplified in *Leishmania*, we further examined the cap-binding activity of the endogenous LeishIF3d tagged *in vivo* by a CRISPR-Cas9 mediated knock-in of the mNeonGreen tag. The tag was inserted between the 5′UTR and the open reading frame of the endogenous LeishIF3d in *L. mexicana* cells (Supplementary Figure S2). To validate the proper assembly of the endogenous mNeonGreen tag LeishIF3d subunit into the LeishIF3 complex, we performed an immunoprecipitation of the LeishIF3 complex over c-Myc agarose beads and analyzed the eluted fractions by LC-MS/MS (Supplementary Figure S3). This analysis verified that 9 subunits of LeishIF3 were co-precipitated with the mNeonGreen tagged LeishIF3d, indicating that it was assembled into the mature LeishIF3 complex (Supplementary Table S2).

**FIGURE 2:**
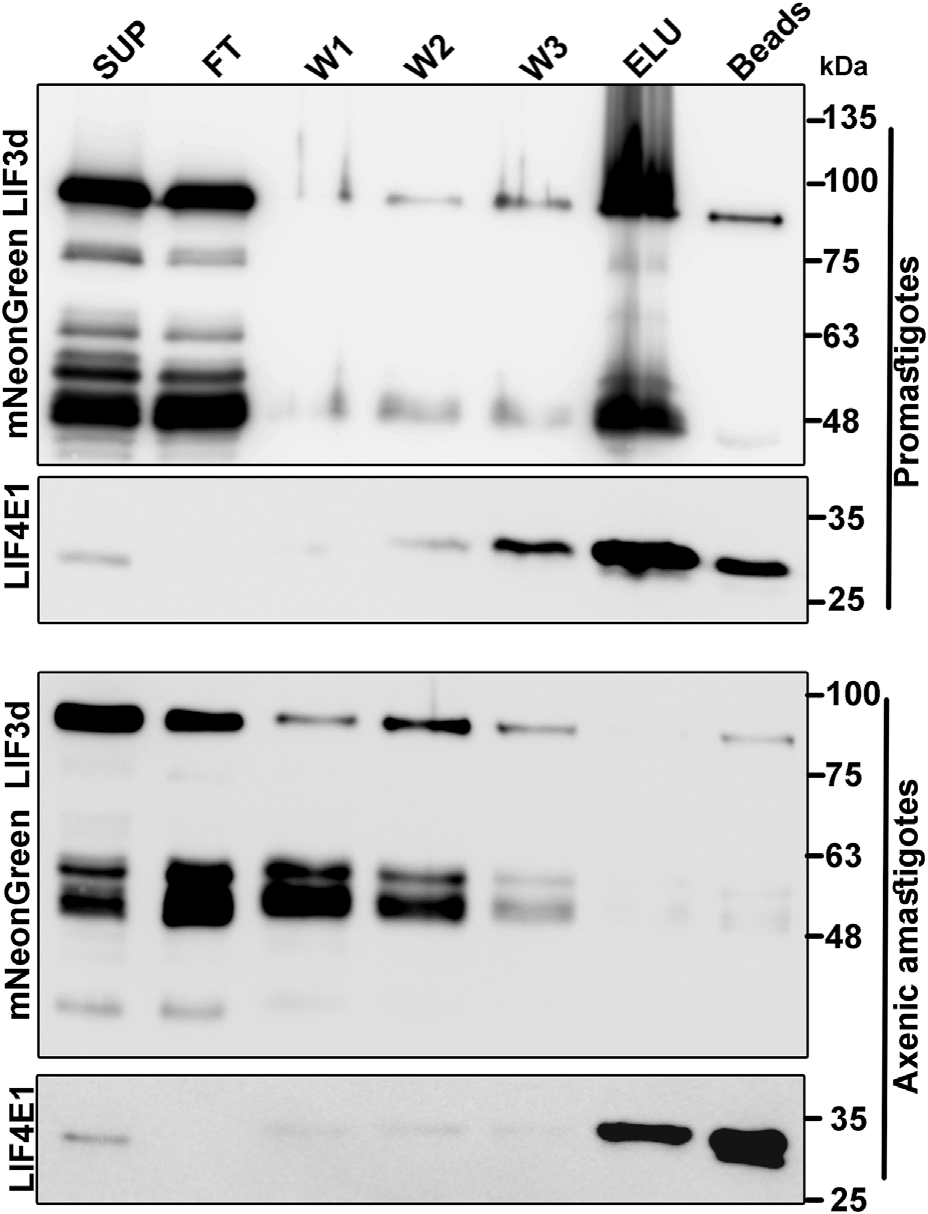
Binding of LeishIF3d to m^7^GTP in the different life forms of *L. mexicana*. Extracts were prepared from promastigotes (10^9^, day2) and axenic amastigotes (10^9^, day 2) in which LeishIF3d was endogenously tagged with mycNeonGreen (mNeonGreen). To monitor binding of the tagged LeishiF3d to m^7^GTP, the extracts were incubated with m^7^GTP-agarose beads. The flow through fraction (FT) was collected, the beads were washed (X3) and further eluted with free m^7^GTP. Following elution, the beads were boiled in SDS-PAGE sample buffer. Aliquots from the soluble extract (SUP, 1%), the flow-through fraction (FT, 1%), the 3 washes (W1-3, 50%), the eluted proteins (ELU, 50%), and the proteins that remained on the beads (50%) were separated over 12% SDS–PAGE and subjected to western blot analysis using antibodies against the Myc tag and antibodies specific for LeishIF4E1.

Based on the finding in mammalian cells that eIF3d can bind the cap structure (Lee et al., 2016), we further investigated whether LeishIF3d could bind the m^7^GTP cap analog in both life stages. Such binding could offer an alternative pathway for translation initiation by bypassing the need for a conventional cap-binding complex (LIF4F), in either life stage. Extracts of mid-log (day 2) promastigote lines in which the endogenous LeishIF3d was mNeonGreen tagged were affinity purified over m^7^GTP-agarose beads. The bound protein complexes were eluted using free m^7^GTP and aliquots from the supernatant, flow through, wash, and the eluted fractions were subjected to western blot analysis using specific antibodies against the Myc-tag and against LeishIF4E1 that served as a positive control for m^7^GTP binding (Figure 2). The cap-binding activity of LeishIF3d to m^7^GTP was most efficient in promastigotes and was dramatically reduced in axenic amastigotes following 4 days of differentiation. Such a reduction was not observed in the ability of LeishIF4E1 to bind m^7^GTP (Figure 2), as this protein was previously reported to actively bind m^7^GTP in both life stages (Zinoviev et al., 2011). The cap-binding activity of LeishIF3d in promastigotes which took place in parallel to the cap-binding activity of different LeishIF4Es could indicate that LeishIF3d is assigned specific tasks. However, the mechanism that differentially regulates the cap-binding activity of LeishIF3d in the different life stages of the parasite requires further investigation. It should also be noted that LeishIF3d appeared to be sensitive to degradation as compared to LeishIF3e and LeishIF4E1. Such degradation prevented us from testing m^7^GTP binding in parasites that were incubated for extended time periods under conditions that induce axenic differentiation.

### Deletion of a single LeishIF3d allele by CRISPR-Cas9 alters the promastigote morphology

**FIGURE 3:**
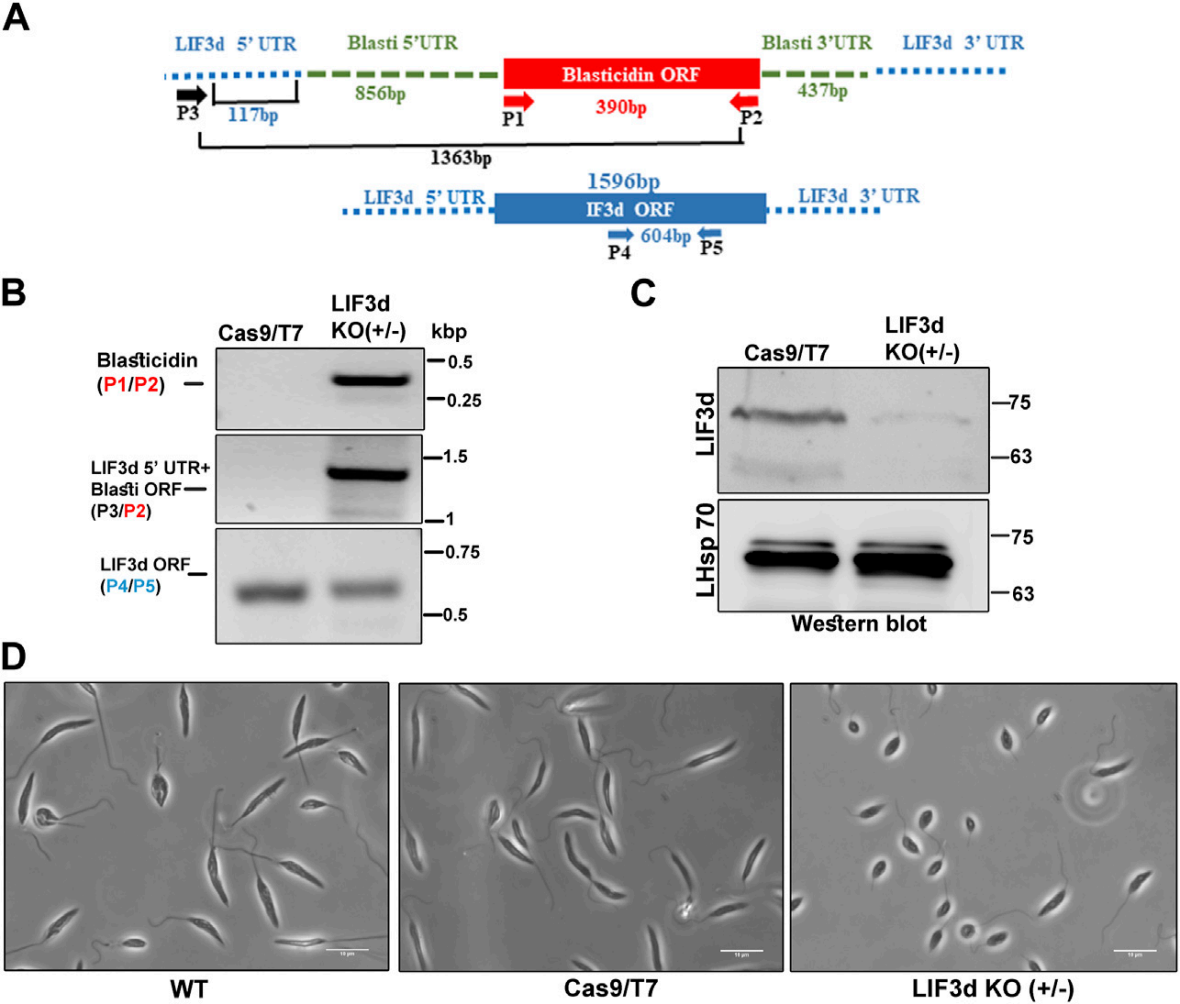
A hemizygous deletion of LeishIF3d(+/−) affects cell morphology. **(A)** A hemizygous deletion of LeishIF3d was generated using CRISPR-Cas9. A schematic representation of the LeishIF3d locus is shown and the primers used for analysis of the deletion are represented by arrows. PCR was applied to test the presence or partial absence of the LeishIF3d gene and to monitor the integration site of the blasticidin resistance marker. Primers derived from the LeishIF3d UTR and from its ORF are shown in black and blue, respectively. Primers derived from the ORF of blasticidin are shown in red. **(B)** Diagnostic PCR was performed to confirm the insertion of the blasticidin resistance gene by replacing a single LeishIF3d allele. Genomic DNA was extracted from the LeishIF3d(+/−) mutant and the *L. mexicana* Cas9/T7 cells. PCR was performed using different combinations of primers derived from the blasticidin ORF (Forward) and Reverse (P1/P2), LeishIF3d 5′ UTR (position −117 bp, Forward) and blasticidin reverse (the product size was 1,363 bp), (P3/P2) and LeishIF3d ORF (P4/P5) the product size was 604 bp. **(C)** Both cell lysates were extracted and resolved over 12% SDS-PAGE, which were subjected to western analysis using an antibody raised against LIF3d. Hsp 70 served as loading control. **(D)** Mid-log phase (Day 2) promastigotes of LeishIF3d(+/−) cells, wild-type (WT), and Cas9/T7 expressing cells from cultures with a similar cell count were fixed with 2% paraformaldehyde and visualized by phase contrast microscopy at ×100 magnification. While WT and Cas9/T7 expressers show an elongated cell morphology and a long flagellum typical of promastigotes, the mutant LeishIF3d(+/−) cells are small, rounded up, and equipped with a short flagellum.

To elucidate the potential role of LeishIF3d during the parasite life cycle, we attempted to delete its two alleles and examine how this deletion affects the parasite phenotype. The *L. mexicana* cell line expressing Cas9 and T7 RNA polymerase was first generated, followed by selection for hygromycin resistance. Specific sgRNAs that targeted LeishIF3d at its 5′and 3′UTRs were transfected into the *L. mexicana* Cas9/T7 expressing line (Shrivastava et al., 2019b). The sgRNAs were designed to cleave around the target gene, promoting its replacement with the blasticidin repair fragment. The LeishIF3d deletion cell line was selected in the presence of blasticidin (20 μg/mL) and a diagnostic PCR analysis indicated that a single allele was eliminated. After confirming the deletion of one allele, we tried to delete the second LeishIF3d allele by replacing it with a G418 repair cassette, but this attempt did not yield viable cells. Diagnostic PCR was performed with the genomic DNA of the mutant cells using several primer pairs (Figure 3A, Supplementary Figure S4A). The presence of the blasticidin selection marker in the genome was verified by primers P1/P2 that were derived from the blasticidin gene, giving a 390 bp product only in the mutant LeishIF3d(+/−) and not in the Cas9/T7 control [Figure 3B (top panel), Supplementary Figure S4B]. Integration of the blasticidin replacement cassette in the proper genomic target site was verified by PCR using primers derived from the LeishIF3d 5′ UTR (P3, forward) and from the open reading frame of the blasticidin resistance gene (P2, reverse); the PCR generated a PCR product of 1,363 bp [Figure 3B (middle panel), Supplementary Figure S4C]. However, the diagnostic PCR using primers (P4/P5) derived from the LeishIF3d open reading frame (ORF) confirmed the presence of an intact LeishIF3d gene copy 604 bp, [Figure 3B (bottom panel), Supplementary Figure S4D]. These two reactions confirmed that one of the two LeishIF3d alleles was deleted, whereas the second allele was still present in the genome. Western blot analysis using antibodies specific for LeishIF3d (Figure 3C), showed that the elimination of a single LeishIF3d allele led to a reduction in the steady-state level of LeishIF3d expression in the LeishIF3d(+/−) mutant cells as compared to the Cas9/T7 control cells, whereas LeishHsp70 remained unchanged.

To examine the morphological changes that were observed in the LeishIF3d(+/−) mutant cells, these cells along with wild-type and Cas9/T7 expressing *L. mexicana* promastigotes were grown at 25°C, washed with PBS and fixed with 2% paraformaldehyde. The slides were visualized by phase contrast microscopy at 100 × magnification. An altered morphology of the mutant LeishIF3d(+/−) cells was observed, where the cells became round and their flagellum length was reduced and deviated from the typical promastigote form (Figure 3D).

### Global translation is reduced by the deletion of a single LeishIF3d allele

**FIGURE 4:**
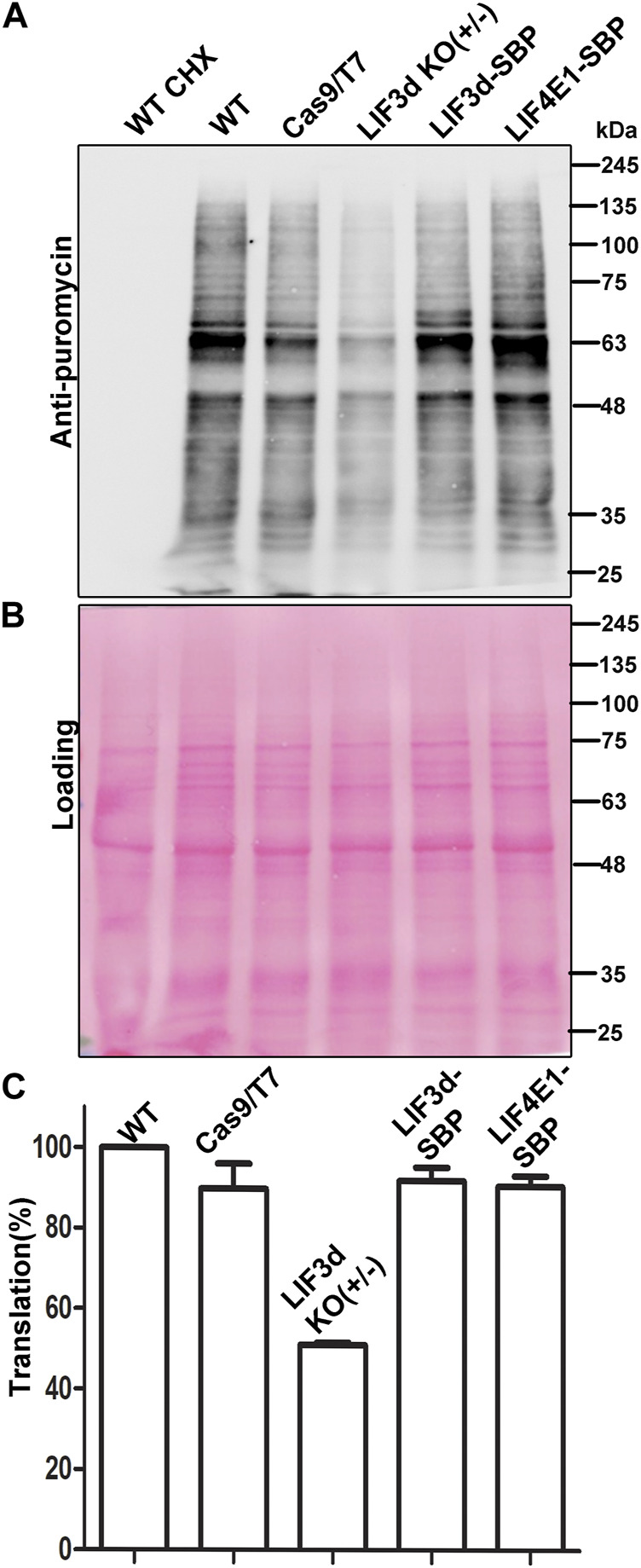
Global translation is reduced in the LeishIF3d(+/−) mutant. Global translation was monitored by incorporation of puromycin into the A site of the ribosome. **(A)** LeishIF3d(+/−) cells, wild-type (WT), Cas9/T7, SBP-tagged LeishIF3d (LIF3d), and LeishIF3e (LIF3e) cells were incubated with 1 μg/mL puromycin for 30 min. Cycloheximide treated cells were used as a negative control for complete inhibition of translation. Puromycin treated cells were lysed in SDS-Laemli buffer, resolved over 10% SDS-PAGE, and subjected to western analysis using antibodies against puromycin. **(B)** Ponceau staining was used to indicate comparable protein loads. **(C)** Densitometry analysis of puromycin incorporation in the different cell lines compared to WT cells (set s 100%). Data from three independent experiments are represented.

To investigate how the reduced level of LeishIF3d expression in the hemizygous mutant affected overall translation, we used the SUnSET assay, which monitors the incorporation of puromycin into the growing polypeptide chains (Goodman and Hornberger, 2013). Puromycin is a structural analogue of amino acyl tRNA that occupies the ribosomal A site. Its integration into the polypeptide chains blocks their elongation and results in translation termination. The puromycin tagged polypeptide chains were examined by western analysis using anti-puromycin antibodies, thereby giving a snapshot of the global translation at a given time point. Mid-log LeishIF3d(+/−) mutant, control WT and Cas9/T7 expressing cells, LeishIF3d-SBP and transgenic cells expression SBP-tagged LeishIF4E1 were incubated with puromycin (1 μg/mL) for 1 h. The cells extracts were resolved on SDS-PAGE and the separated proteins were subject to western analysis using antibodies against puromycin, and Ponceau Staining signified the equal loading of each lane (Figure 4B). The cycloheximide treated sample served as a negative control with no active puromycin incorporation. LeishIF3d(+/−) mutant cells show approximately 50% reduction in global translation as compared to control WT and Cas9/T7 expressing cells (Figure 4A). Translation was not affected in the LeishIF4E1 control cells, as well as in the LeishIF3d over-expressing cells. Densitometric analysis showed a significant decrease in the relative translation rate of the mutant cells (50%) as compared to wild-type (WT, 100%), Cas9/T7-expressing cells (∼89%), LeishIF4E1-SBP expressers (∼90%), and LeishIF3d-SBP expressers (∼91%) (Figure 4C). We therefore concluded that deletion of a single LeishIF3d allele affected global translation in *Leishmania*. The decreased translation in the mutant LeishIF3d(+/−) cells was also supported by monitoring the polysome levels in sucrose gradients. A lower amount of polysomes was observed in the mutant LeishIF3d(+/−) cells, as compared to the control cells that expressed Cas9/T7 (Supplementary Figure S5).

### Proteomic analysis of the LeishIF3d(+/−) mutant cells shows a downregulation of proteins involved in specific biological processes

**FIGURE 5:**
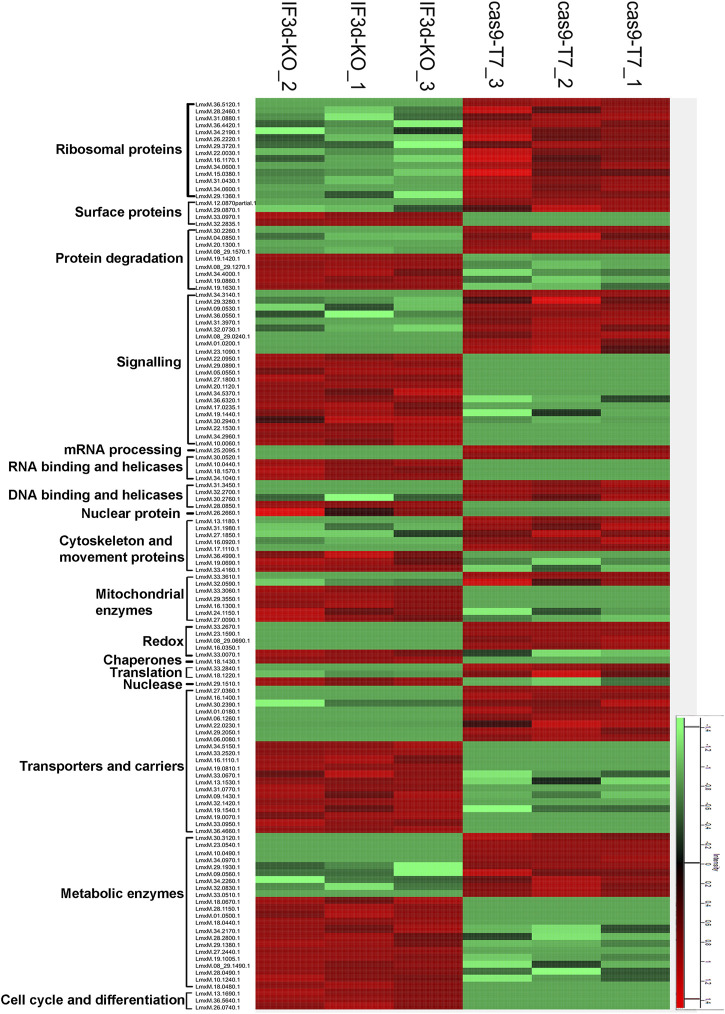
The hemizygous deletion of LeishIF3d alters the proteomic profile of the LeishIF3d(+/−) cells. The proteomic content of LeishIF3d(+/−) and Cas9/T7 cells was determined in triplicates by LC-MS/MS. The heatmap displays the intensities of differentially expressed proteins in the LeishIF3d mutant by at least log2 = 1 as compared to Cas9/T7 cell extracts, with q < 0.05. Protein intensities were normalized by z-score. The color scale illustrates the relative expression level of each protein across the 3 repeat samples; red and green indicate higher and lower expression, respectively. The different columns show the three biological repeats of each sample; the rows represent the individual proteins.

To examine the effect of the hemizygous deletion of one of the two LeishIF3d alleles on the proteomic profile, we carried out a mass spectrometry analysis of the total cell lysate derived from the LeishIF3d(+/−) mutant cells, as compared to Cas9/T7 control cells. This analysis was performed with three independent samples that were analyzed in parallel and in the same run. The resulting peptides were identified by their comparison to the annotated *L. mexicana* genome in TriTrypDB, and these were further quantified by the MaxQuant software. The statistical analysis was performed by the Perseus software platform (Tyanova et al., 2016). The intensity for each protein that was significantly up or downregulated in the LeishIF3d(+/−) mutant cells and Cas9/T7 control cells is shown for each biological repeat as a heat map (Figure 5).

The raw data of the MS/MS analysis contained 1752 proteins (Supplementary Table S3). We categorized the proteins that were up or downregulated in the LeishIFd3d(+/−) cells as compared to the Cas9/T7 control by at least log2 = 1, based on their function (Supplementary Figure S6A). The statistical analysis highlighted the downregulation of 100 proteins and upregulation of 159 proteins (Supplementary Table S3). The pie diagram shows a reduction in ribosomal proteins and proteins related to ribosome biogenesis (Supplementary Figure S6A). A major decrease (log2 = 10.7) was observed for LmxM.36.5120.1, a protein that is part of the 40S ribosomal subunit. Changes in other ribosomal proteins that were part of the 60S subunit showed a much lower decrease, that ranged at ∼ log2 = 1. A log2 = 5.2- decrease in the protein associated with mRNA capping (Capping enzyme RNA triphosphatase (LmxM.25.2095) strengthens the association of LeishIF3d with mRNA metabolism. The reduction in cytoskeletal and movement proteins was in line with our microscopy data that showed alterations in the morphology and flagellar length of the LeishIF3d(+/−) promastigote cells (Supplementary Table S3). Other downregulated proteins in the LeishIF3d(+/−) proteome included metabolic and transporter proteins. Upregulation was also observed for proteins that were associated with other biological processes such as signaling, cell cycle progression and protein degradation (Supplementary Table S3).

The downregulated proteins were also evaluated by the Gene Ontology (GO) enrichment analysis through the TriTrypDB platform, based on their biological processes. Supplementary Figure S6B and Supplementary Table S4 highlight the major categories of the downregulated protein groups, each containing at least two proteins. In line with the manually categorized proteins, the GO enrichment analysis also showed that the downregulated proteins related to translational activity. The GO enrichment also denotes a downregulation in enzymes involved in tRNA processing, cellular amino acid catabolic processes, and peptide biosynthesis (Supplementary Figure S6B).

Figure 5 shows a heatmap of proteins that had a change of at least log2 = 1 as compared to controls with an adjusted q value of <0.05 were categorized into functional groups (Supplementary Table S3). The manual classification highlighted changes in proteins encoding ribosomal proteins, cytoskeletal proteins, and several RNA binding proteins as well as signaling, chaperone, cytoskeletal, and surface proteins. The proteins are also shown in a heatmap that contains hierarchical clustering (Supplementary Figure S7).

### Disruption of the α helices 5 and 11 affects the growth and cap binding affinity of LeishIF3d

**FIGURE 6:**
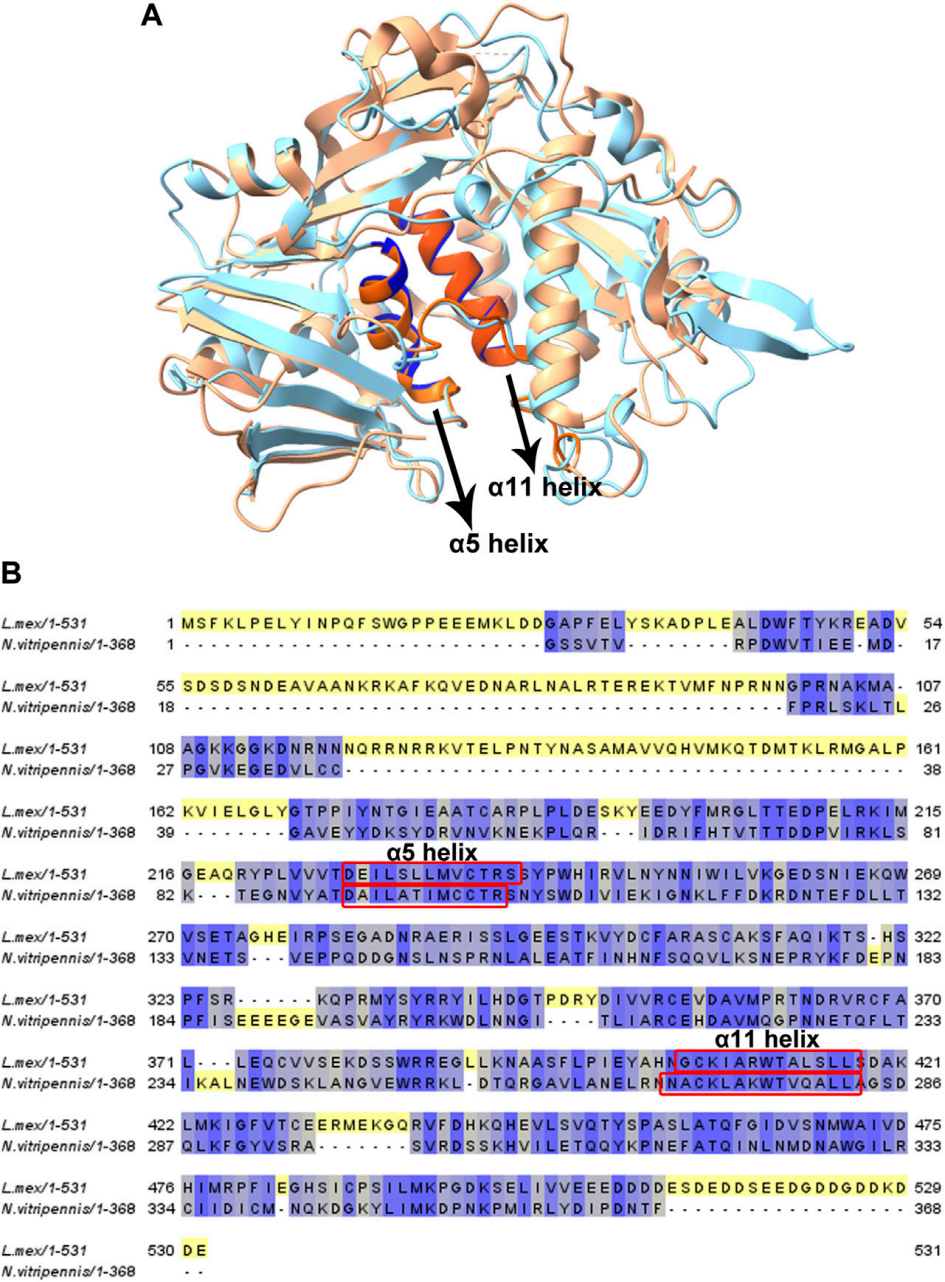
Homology modeling and sequence alignment of LeishIF3d with *Nasonia sp*. IF3d. **(A)** Homology modeling of LeishIIF3d _(142–504)_ was carried out using AlphaFold. All figures were generated using ChimeraX 1.2.5. A Superposition of the LeishIF3d model structure, IF3d (*Nasonia vitripennis*; PDB code 5K4B) was performed. Light blue represents *Leishmania* IF3d, and orange represents *Nasonia vitripennis* IF3d structure. α helix 5 and α helix 11 are highlighted in bold colors. **(B)** The alignment includes sequences of IF3d from *Leishmania mexicana*, and *Nasonia vitripennis*. Alignment was generated using Jalview (2.10.5). The sequences were subjected to sequence alignment to highlight their homologies. 2 helices which are responsible for cap-binding are highlighted in red box.

eIF3 has a role in each step of translation initiation, including ribosome loading, scanning, and start codon selection (Valasek et al., 2017). A previous study revealed that eIF3d serves as an alternative mRNA cap-binding protein that was responsible for transcript-specific translation, and two conserved helices (α5 and α11) were shown to be involved in cap recognition (Lee et al., 2016). Figure 6A shows a homology model of LeishIF3d (blue) that was obtained from the online AlphaFold server. Images were prepared via ChimeraX 1.2.5. The predicted structure was then superposed on the eIF3d from Nasonia *vitripennis* (PDB: 5K4B, orange) by ChimeraX 1.2.5. The superposed model confirms the presence of two alpha helices, as shown in the N. *vitripennis* structure. These were alpha helix 5 and alpha helix 11, that extended between residues 228–239 and 405–417, respectively. The predicted alpha helix structures in LeishIF3d were aligned with the parallel alpha helices in the N. *vitripennis* structure. The sequences of both alpha helices were also aligned with each other (Figure 6B). The presence of the two alpha helices was also observed when the structure of LeishIF3d was predicted by Swiss PDB, that gave a very similar prediction (Supplementary Figure S8).

**FIGURE 7:**
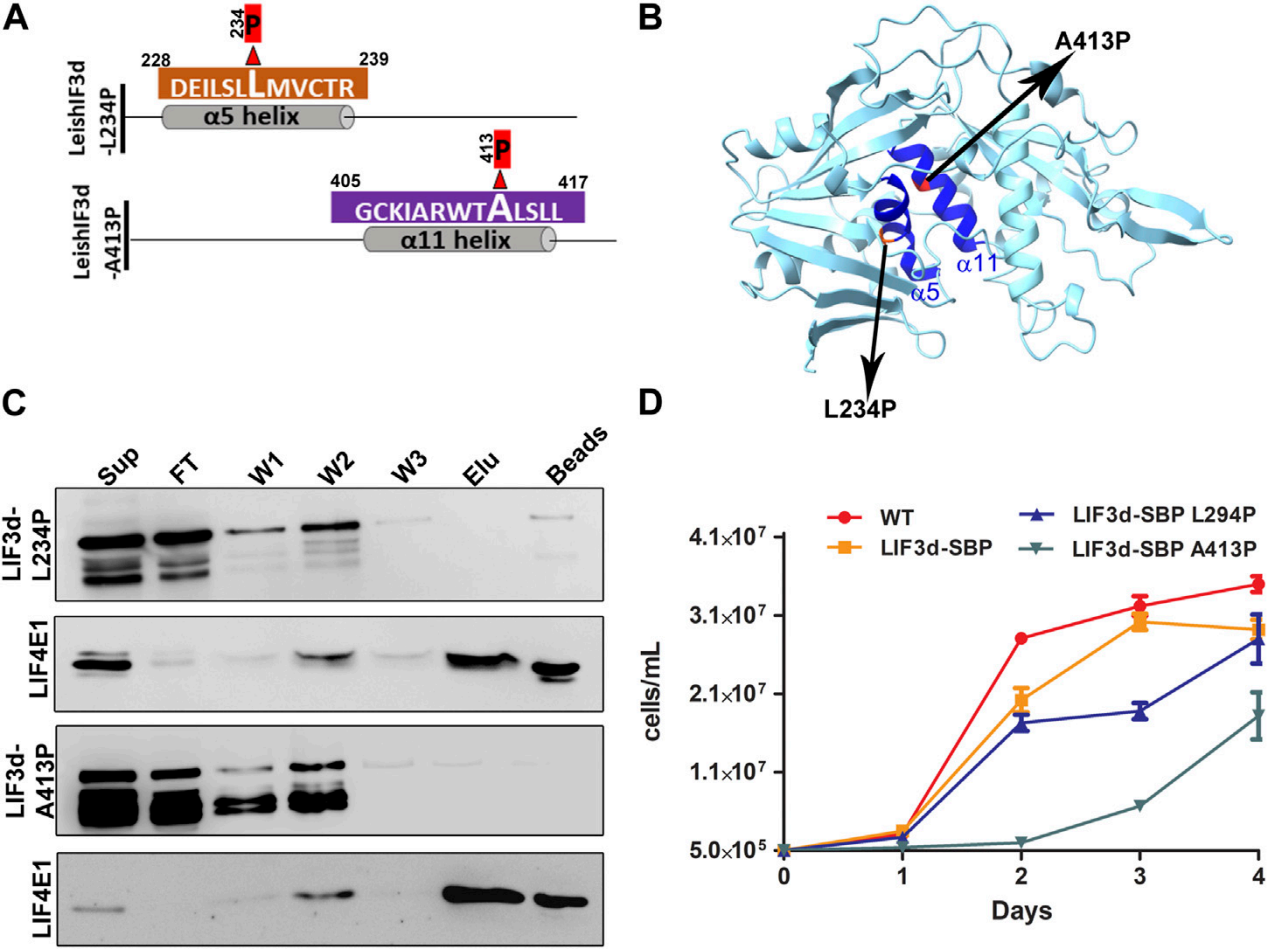
Disruption of α helix 5 and α helix 11 affects growth and cap-binding activity of LeishIF3d. **(A)** A schematic diagram showing a single amino acid change in each α helix 5 and α helix 11 of LIF3d. To disrupt each of the α helices, two mutants were generated, one in which a proline residue replaced lysin 234 in α helix 5 and another in which the proline replaced the alanine residue at position 413 in α helix 11. **(B)** The predicted 3-D structure of a LeishIF3d_(142–504)_ was generated using ChimeraX 1.2.5. The alpha helices 5 and 11 are highlighted in dark blue. The two mutated residues in α helix 5 and α helix 11 are highlighted. **(C)** Cell extracts (from 10^9^ cells) obtained from promastigotes expressing the episomal SBP-tagged LeishIF3d L234P and LeishIF3d A413P were incubated with m^7^GTP-agarose beads. The beads were washed and further eluted with free m^7^GTP. Aliquots of the soluble extract (SUP, 1%), flow-through (FT, 1%), the three washes (W1-3, 50%), the eluted fraction (ELU, 50%) and the proteins that remained on the beads (50%), were separated over 12% SDS–PAGE. Blots were analyzed using antibodies against the streptavidin binding protein tag (SBP) and antibodies specific for LeishIF4E1 (LIF4E1). **(D)** Growth curves of the different cell lines were generated by monitoring the cell counts during five consecutive days. The curves were obtained from three independent assays, bars representing standard deviations are also marked. Wild-type (WT) cells are shown in red, cells over-expressing LeishIF3d are shown in yellow, cells over-expressing LeishIF3d L234P are in blue and cells over-expressing LeishIF3d A413P are in green.

To validate that these two helices have a role in the cap binding activity in *Leishmania*, we introduced a mutation that exchanged the leucine at position 234, in the middle of the alpha helix 5, with a proline residue. In alpha helix 11, we exchanged the alanine at position 413 with a proline residue. Introducing a proline residue in the alpha helix is known to disrupt such structures (Li et al., 1996; Nilsson et al., 1998; Kumeta et al., 2018) and could therefore indicate whether this structure was required for the cap-binding activity. We then generated transgenic cell lines expressing the two mutant proteins, LeishIF3d (L234P) with the mutation in alpha helix 5 and LeishIF3d (A413P) with the mutation in alpha helix 11, as SBP-tagged LeishIF3d proteins (Figures 7A, B). Extracts of LeishIF3d-(L234P) and LeishIF3d (A234P) were affinity purified over m^7^GTP-agarose beads that were eluted with free m^7^GTP. Binding was monitored in the eluted fractions. Western analysis indicated that indeed the SBP-tagged mutated LeishIF3d L234P and A234P proteins showed decreased binding to m^7^GTP as compared to the positive binding control of LeishIF4E1 (Figure 7C). This disrupted cap-binding activity could suggest that the alpha 5 and alpha 11 of LeishIF3d are important for binding to m^7^GTP.

We further investigated the effect of expressing LeishIF3d with disrupted helices alpha 5 and alpha 11 on parasite growth. We found that the proliferation rate of the LeishIF3d-L234P and LeishIF3d-A413P mutant promastigotes was significantly slower as compared to the growth of control wild-type cells, or cells expressing transgenic SBP-tagged LeishIF3d (Figure 7D).

### Recombinant LeishIF3d competes with LeishIF4E1 for binding to m^7^GTP

**FIGURE 8:**
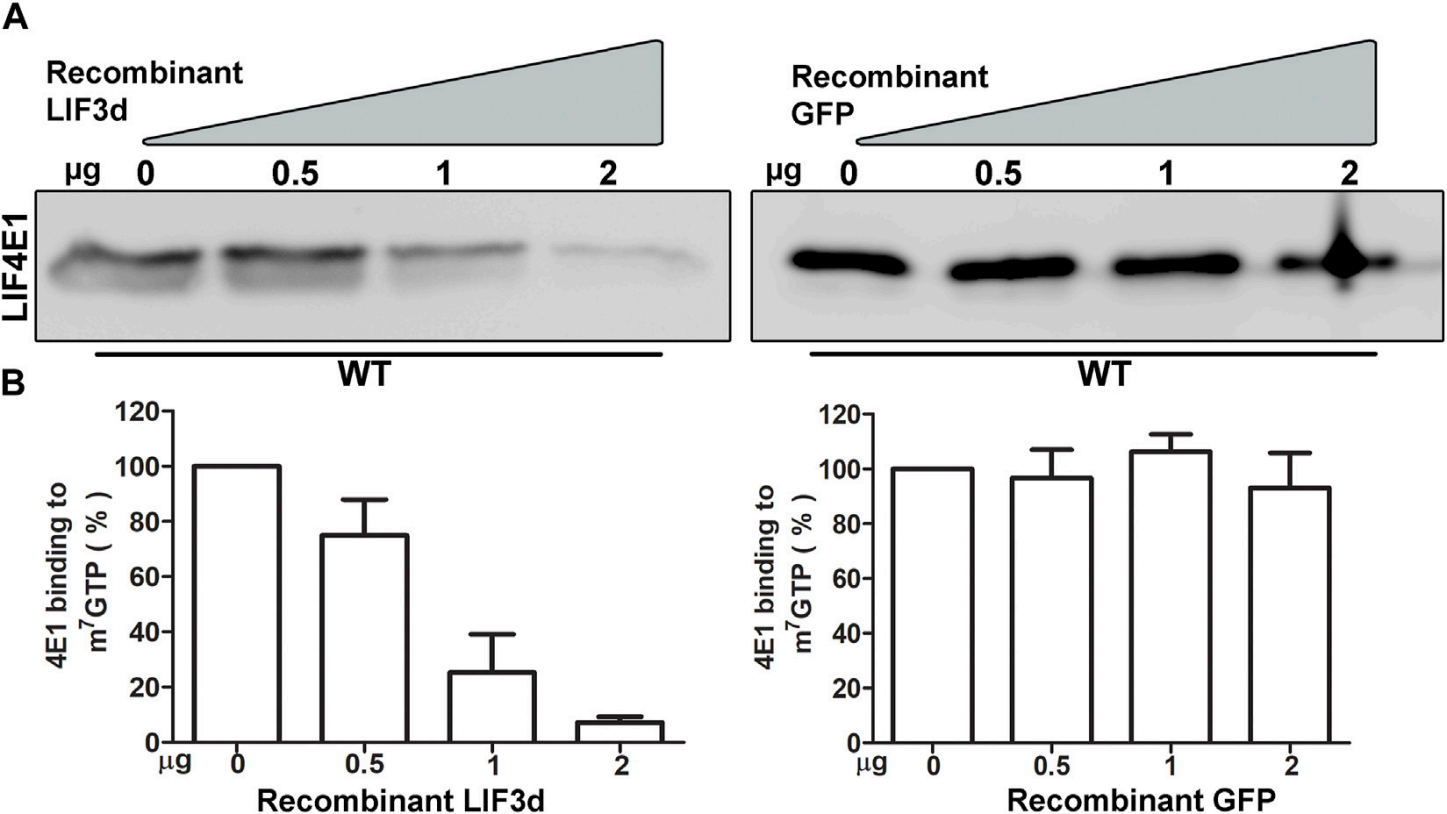
Competition between Recombinant LeishIF3d and the endogenous LeishIF4E1 over m^7^GTP binding **(A)** Cell extracts from wild-type *Leishmania mexicana* promastigotes were affinity-purified over m^7^GTP agarose beads in the presence of increasing concentrations of purified recombinant His tagged LeishIF3d, or in the presence of recombinant GFP as a control. Following the washes, the m^7^GTP-agarose beads were boiled in sample buffer, to release the LeishIF4E1 that remained bound to the resin. The eluted proteins were separated on SDS-PAGE and subjected to western analysis using antibodies against LeishIF4E1 (LIF4E1). **(B)** Densitometry analysis of the blots (bottom panel) shows that the binding of LeishIF4E1 to m^7^GTP was reduced in the presence of increasing amounts of LIF3d in a dose-dependent manner (0 μg, set as 100% signal). Such a reduction was not observed for GFP. The data show the mean of three independent experiments. Error bars indicate the standard error.

Deciphering the roles of the multiple LeishIF4E paralogs is a challenging goal. They vary in their relative expression and cap-binding activity. The cap-binding activity of LeishIF4E1 is efficient in both life forms, as shown in our *in vitro* studies using promastigotes and axenic amastigotes and in promastigote (Zinoviev et al., 2011). In search of alternative cap-binding proteins, we show here that LeishIF3d, a subunit of the LIF3 translation factor, can independently bind the cap structure. To understand whether LeishIF3d and LeishIF4E1 compete, we examined the cap-binding activity of endogenous LeishIF4E1 in the presence or absence of recombinant His-tagged LeishIF3d (Supplementary Figure S1) that was added in a dose dependent manner. *L. mexicana* wild-type cells were incubated with increasing concentrations of recombinant and purified His-LeishIF3d (0, 0.5, 1, 2 µg), or with purified GFP alone (0, 0.5, 1, 2 µg) as control. The beads were then washed three times and eluted by boiling in SDS-Laemmli’s buffer; the eluted proteins were subjected to western analysis. Figure 8A shows that inclusion of LeishIF3d in the incubation mixture with LeishiF4E1 reduced the cap-binding activity of LeishIF4E1 in a dose-dependent manner. Densitometry analysis revealed that at the lowest dose of LeishIF3d, the cap-binding activity of LeishiF4E1 reduced to 70% of that monitored in its absence of LeishIF3d, whereas increasing the LeishIF3d dose showed a reduction to 30% and 10% (Figure 8B). We observed no effect upon the addition of a recombinant GFP protein (negative control). Our results show that LeishIF3d is a potential competitor of LeishIF4E1 for binding to the cap structure.

## Discussion

*Leishmania* parasites encode a complex network of cap-binding proteins that generate multiple unique complexes. These are assumed to assist the parasites in their adaptation to changing environmental conditions whereby translation regulation plays a key role. To investigate the translation apparatus of *Leishmania* we have been studying the different cap-binding complexes in this organism. There are six cap-binding protein paralogs, of which only four have an eIF4G binding partner. These complexes are formed and are functional mainly in the promastigote life form, except for LeishIF4E1, which in addition to its efficient binding to m^7^GTP in promastigotes, it is the only cap-binding protein that continues to bind m^7^GTP in axenic amastigotes. In contrast to LeishIF4E1, the other LeishIF4E paralogs lose their activity under conditions of elevated temperatures and reduced pH (Zinoviev et al., 2011; Shrivastava et al., 2021). We therefore considered the possibility that translation in amastigotes follows a non-canonical path, and examined additional routes by which translation initiation can proceed in amastigotes. Recent reports in mammalian cells highlighted a cap-binding activity to eIF3d, that drives translation in a transcript-specific manner (Lee et al., 2016). Here we examined whether LeishIF3d could serve as a driving force for translation initiation in promastigotes and amastigotes.

IF3d is a part of a multi-subunit complex eIF3 (Wagner et al., 2016; des Georges et al., 2015; Zhou et al., 2008; Wagner et al., 2014). eIF3 participates in assembly of the preinitiation complex of translation over the cap-structure but is also known to be involved in cap-independent translation (Wolf et al., 2020). eIF3 has a significant role in translation initiation, termination, and in ribosomal recycling (Gomes-Duarte et al., 2018). Despite the large evolutionary distance between *Leishmania* and other eukaryotes, the parasite LeishIF3 complex shows a compositional similarity (Meleppattu et al., 2015), with the parasite complex containing subunits a-l, and canonical interactions such as between subunits “a” and “c” were validated. Based on a previous report in mammalian cells showing that the “d” subunit of eIF3 can bind m^7^GTP, we examined whether the *Leishmania* ortholog LeishiF3d could also bind the cap-structure, and if so whether it could offer a novel route of translation initiation in this parasite.

In this study we show that the parasite LeishIF3d subunit indeed binds m^7^GTP. This was first shown using affinity chromatography with extracts of cells that express transgenic tagged LeishIF3d. The individual binding of LeishIF3d to m^7^GTP was further validated using the recombinant LeishIF3d, thus excluding the possibility that the binding of endogenously tagged LeishIF3d could be mediated through other proteins that are present in the complex. However, binding of LeishIF3d to m^7^GTP could be observed only in promastigotes. Unlike in promastigotes, cell lysis of axenic amastigotes led to impaired binding of LeishIF3d. This could indicate that LIF3d went through some changes in its interacting partners or that it went through conformational changes under conditions that induce axenic differentiation. The latter possibility is in line with the altered sensitivity to degradation, observed in amastigotes. The increased sensitivity to proteolytic cleavage during *Leishmania* cell lysis was previously reported for Leish4E-IP1 (Meleppattu et al., 2018) and Leish4E-IP2 (Tupperwar et al., 2020). LeishIF4E1 however, was unchanged under these conditions. It keeps its binding capacity and remains stable.

LeishIF3d participates in assembly of the LeishIF3 complex. However, its additional roles in translation have not yet been explored. To further understand its function, we generated a deletion mutant, in which we succeeded in eliminating one of the two LeishIF3d alleles using CRISPR-Cas9 (Beneke et al., 2017). Deletion of a single allele resulted in decreased expression of LeishIF3d, and this had an inhibitory effect on global translation of the parasite, as well as on its morphology and proteomic profile. The promastigote cells were rounded and their flagellum was shorter than usual. We therefore concluded that a threshold level of IF3d is required to maintain its biological and molecular function under normal conditions. The reduced translation was accompanied by decreased expression of proteins that were associated with translation, as indicated by the proteomic profile of total proteins in LeishIF3d(+/−) cells.

A strong decrease was observed in expression of the capping enzyme RNA triphosphatase (LmxM.25.2095.1-p1). This enzyme is known to initiate the capping of mammalian pre-mRNAs and is also functional in trypanosomatids (Ruan et al., 2007). Reduced expression of this enzyme is expected to disturb the biosynthesis of the Spliced Leader RNA, and hence reduce global translation. The proteomic profile of LeishIF3d(+/−) revealed a reduction in proteins that are associated with protein synthesis. This included proteins such as the SA ribosomal protein that is also known as a precursor of the cell-surface laminin receptor, LAMR. In addition to its binding to the 40S ribosomal subunit, SA has a carboxy-terminal domain that is responsible for the binding of laminin as well as prions and several viruses (Malygin et al., 2011). Reduced expression was also recorded for the selenocysteine-specific elongation factor, which was previously reported to be a structural chimera of the Elongation and Initiation factors (Leibundgut et al., 2005). Selenocystein is found mainly in the active site of oxidoreductases, where it is directly involved in catalysis. Unlike cysteine, selenocysteine is negatively charged at physiological pH and highly reactive (Leibundgut et al., 2005). In accordance with this reduction, expression of a redox-related protein, NADPH-cytochrome p450 reductase-like protein (LmxM.33.2670.1) was also recorded.

In view of the reduced translation activity in the LeishIF3d(+/−) mutant, we observed that protein degradation enzymes were also reduced, including a putative calpain-like cysteine peptidase (Ennes-Vidal et al., 2021) and a putative aminopeptidase (Morty and Morehead, 2002). Both are abundant degradation enzymes in these parasites, although their targets and exact roles were not defined yet. The morphological changes observed for the LeishIF3d(+/−) cells are in accordance with the reduced expression of the dynein light chain (LmxM.13.1180), and Kinesin (LmxM.17.1110). These regulate ciliary movement in *Trypanosoma brucei* (Godar et al., 2022) and are essential for cell morphogenesis, cell division, and virulence (Corrales et al., 2021). Reduced expression was recorded also for proteins that encode a variety of metabolic enzymes. These enzymes are known to associate with translation initiation complexes (Soumya et al., 2015; Soares-Silva et al., 2016; Moitra et al., 2019), although their exact role is yet not clear; moonlighting functions could be considered.

Homology modeling of the parasite LeishIF3d shows a strong structural resemblance to the mammalian eIF3d. This includes a region that resembles the cap-binding pocket of eIF4E and is also in accordance with the ability of LeishIF4E1 to compete with the binding of LeishIF3d to m^7^GTP. The homology modeling highlighted two alpha helices adjacent to the cap-binding pocket, alpha 5 and alpha 11, that were shown to be important for the cap-binding activity in the mammalian protein (Lee et al., 2016). Introducing a mutation in which a proline residue replaced the 234 leucine in alpha 5 and the alanine at position 413 in alpha 11 in LeishiF3d, inhibited the binding activity of the mutated LeishIF3d to m^7^GTP. Since proline is known to interfere with the formation of alpha helix structures (Li et al., 1996), this further highlighted the importance of these two elements in the binding activity of LeishIF3d.

This study shows that *Leishmania* has adapted alternative pathways for protein synthesis, based on the cap-binding activity of LeishIF3d. However, our data show that this activity is restricted to promastigotes and cannot account for driving translation in amastigotes. Based on our previous findings and on the data presented in this study, we propose that translation in amastigotes may proceed in a cap-independent pathway.

## Data Availability

The datasets presented in this study can be found in online repositories. The names of the repository/repositories and accession number(s) can be found below: ProteomeXchange Consortium via the PRIDE partner repository with the dataset identifier PXD040501.
