# Supplementary figures and images for: LeishIF3d is a non-canonical cap-binding protein in Leishmania
Source: Front Mol Biosci. 2023 May 30;10:1191934. doi: 10.3389/fmolb.2023.1191934 (PMC10266417; doi:10.3389/fmolb.2023.1191934)

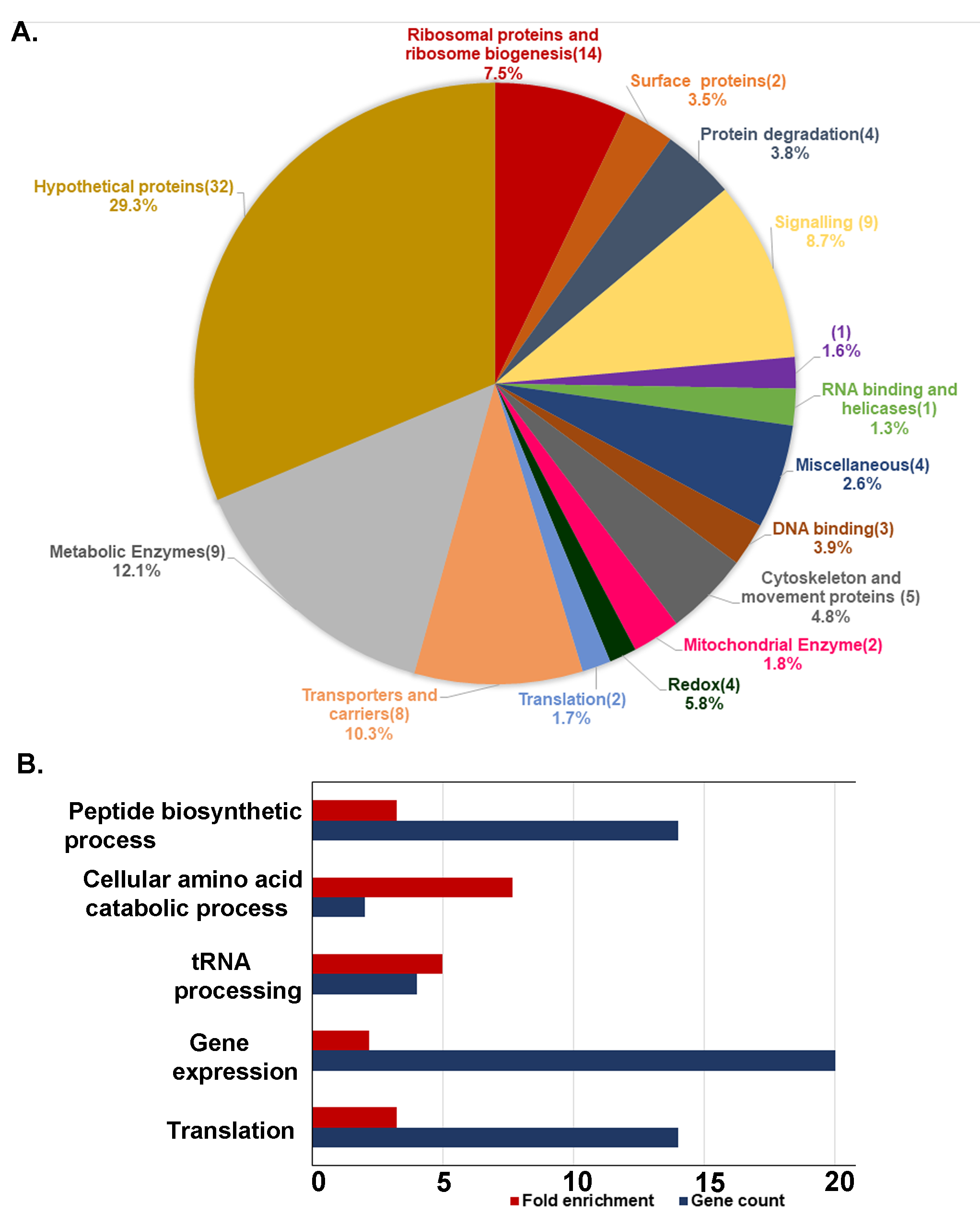

Supplement: Supplementary file 3 [file Image6.TIF]

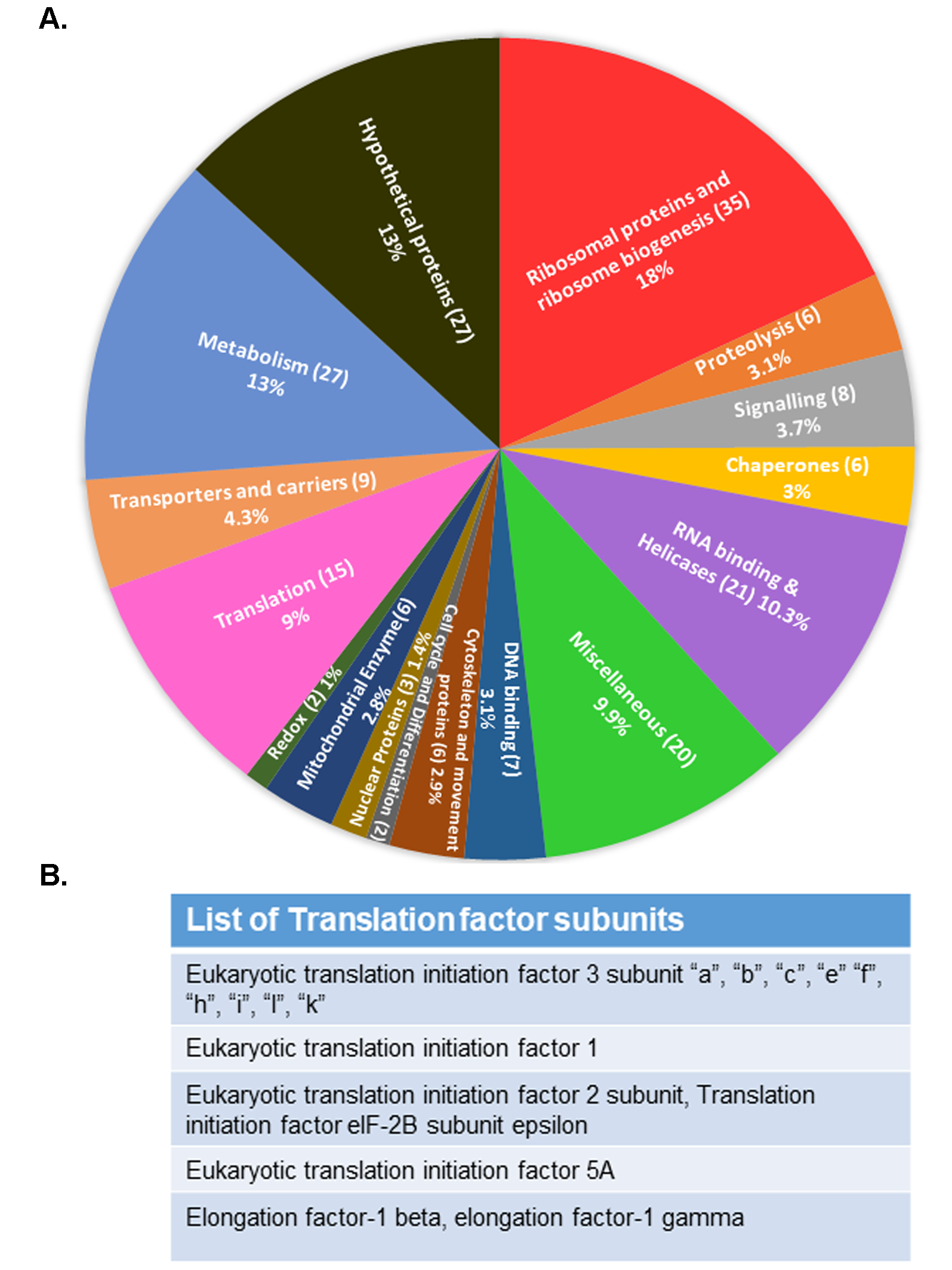

Supplement: Supplementary file 5 [file Image3.TIF]

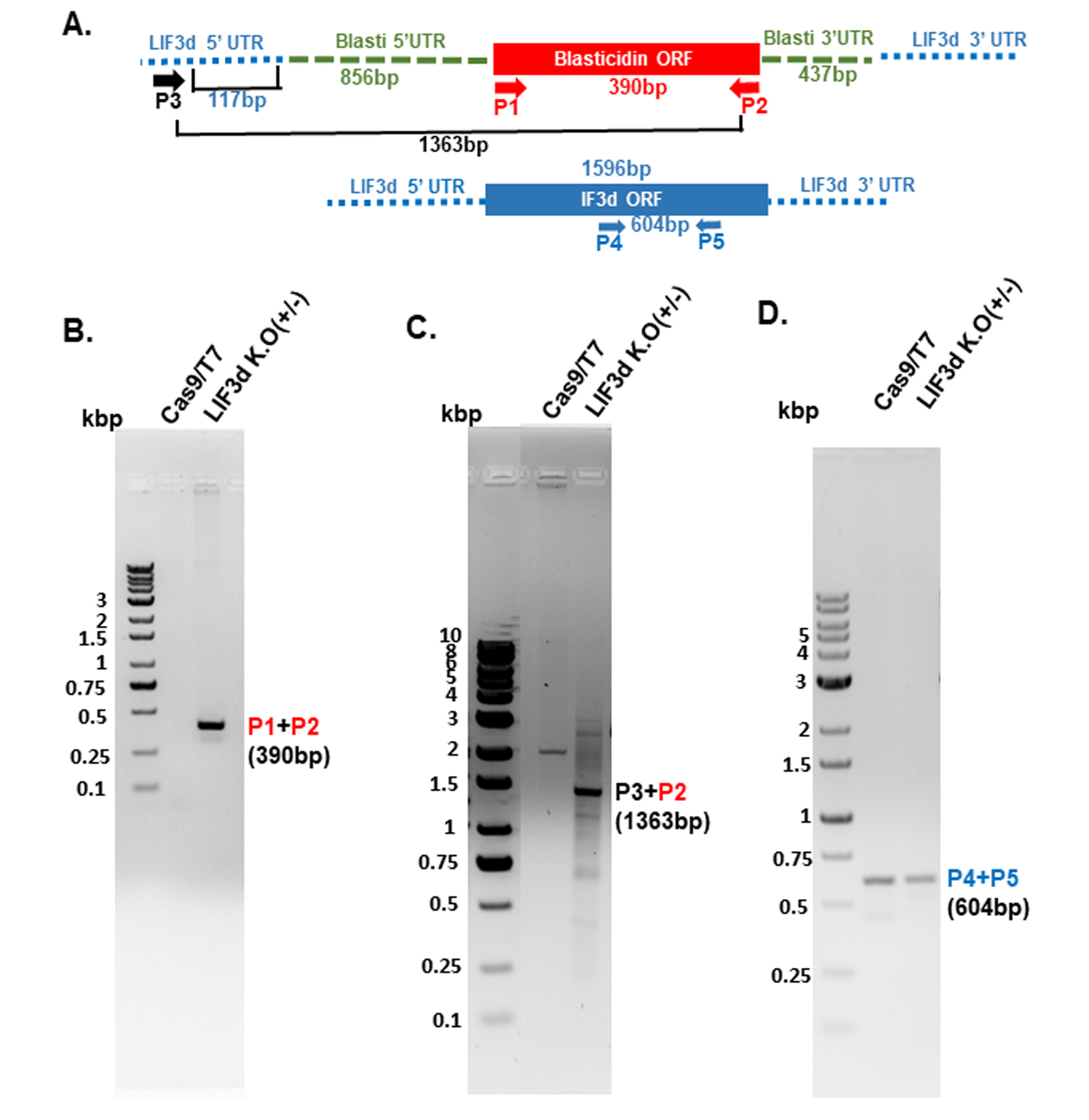

Supplement: Supplementary file 6 [file Image4.TIF]

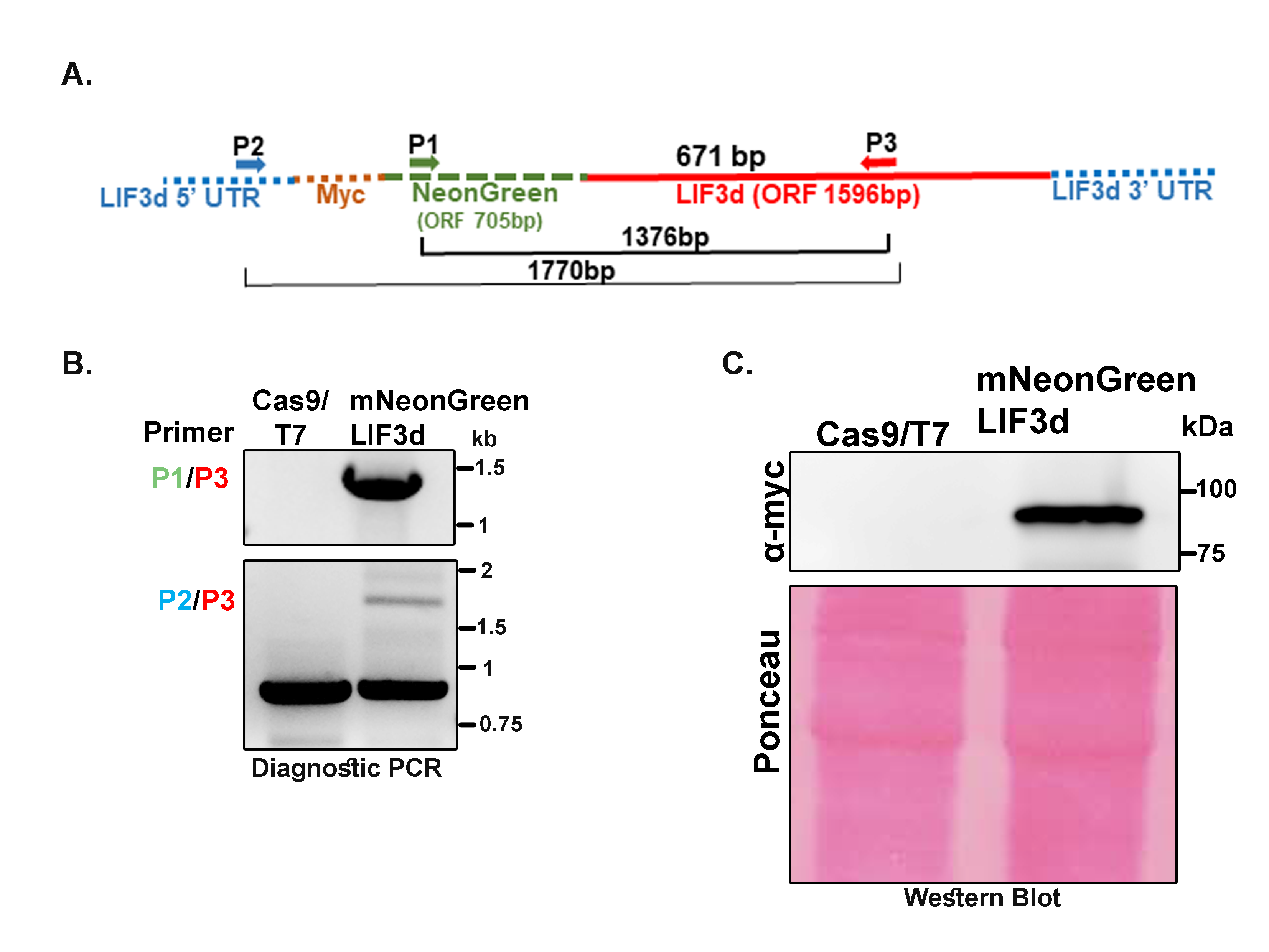

Supplement: Supplementary file 7 [file Image2.TIF]

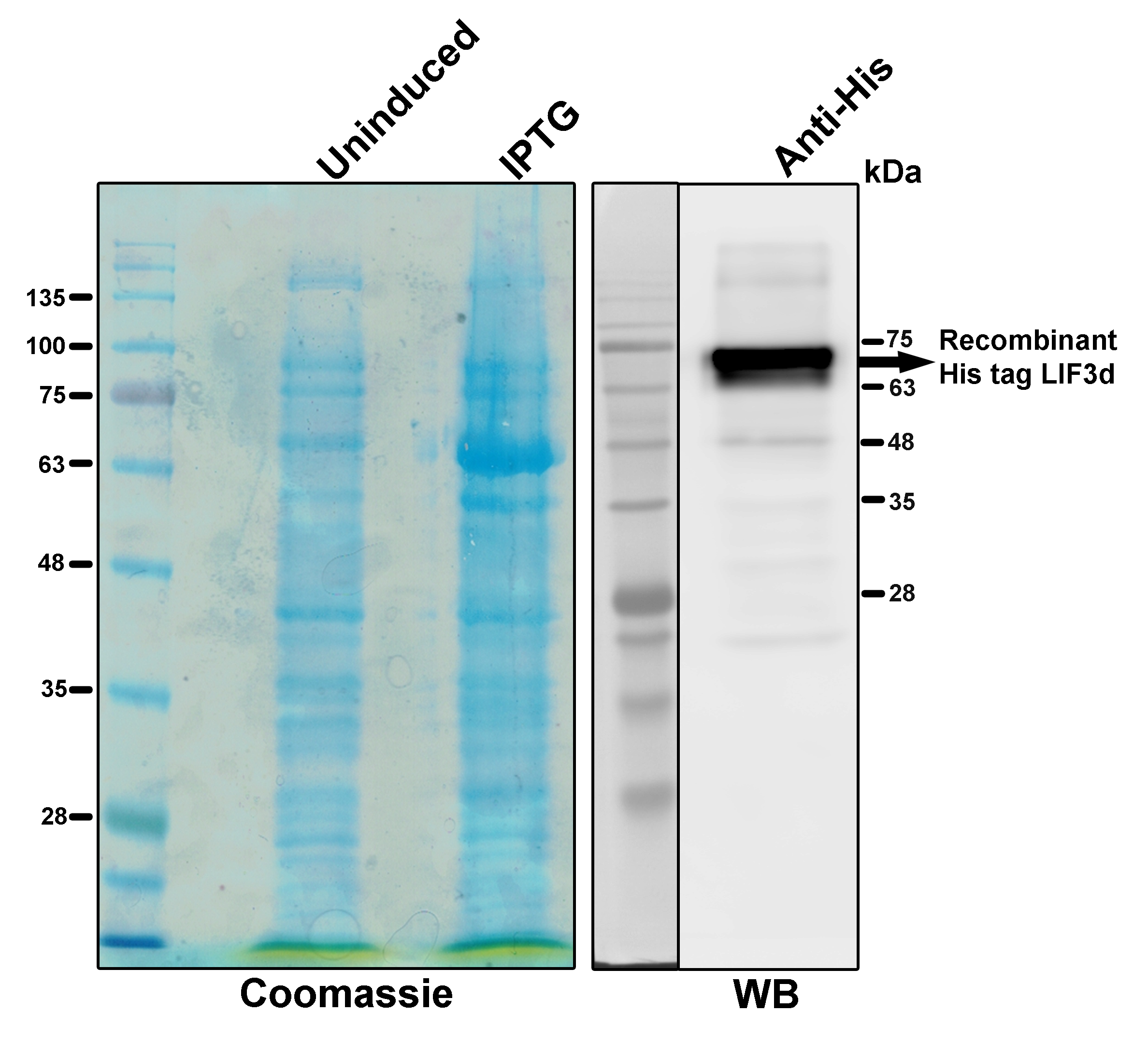

Supplement: Supplementary file 8 [file Image1.TIF]

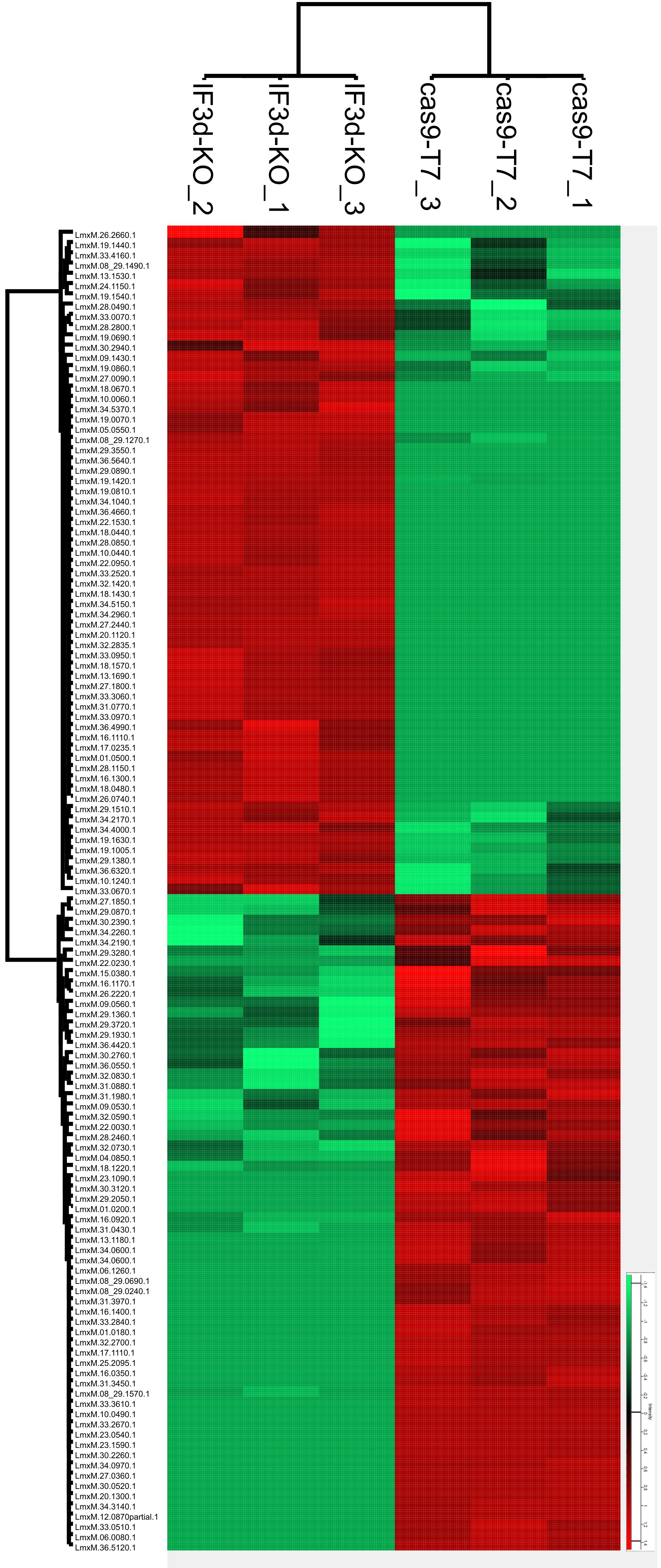

Supplement: Supplementary file 9 [file Image7.TIF]

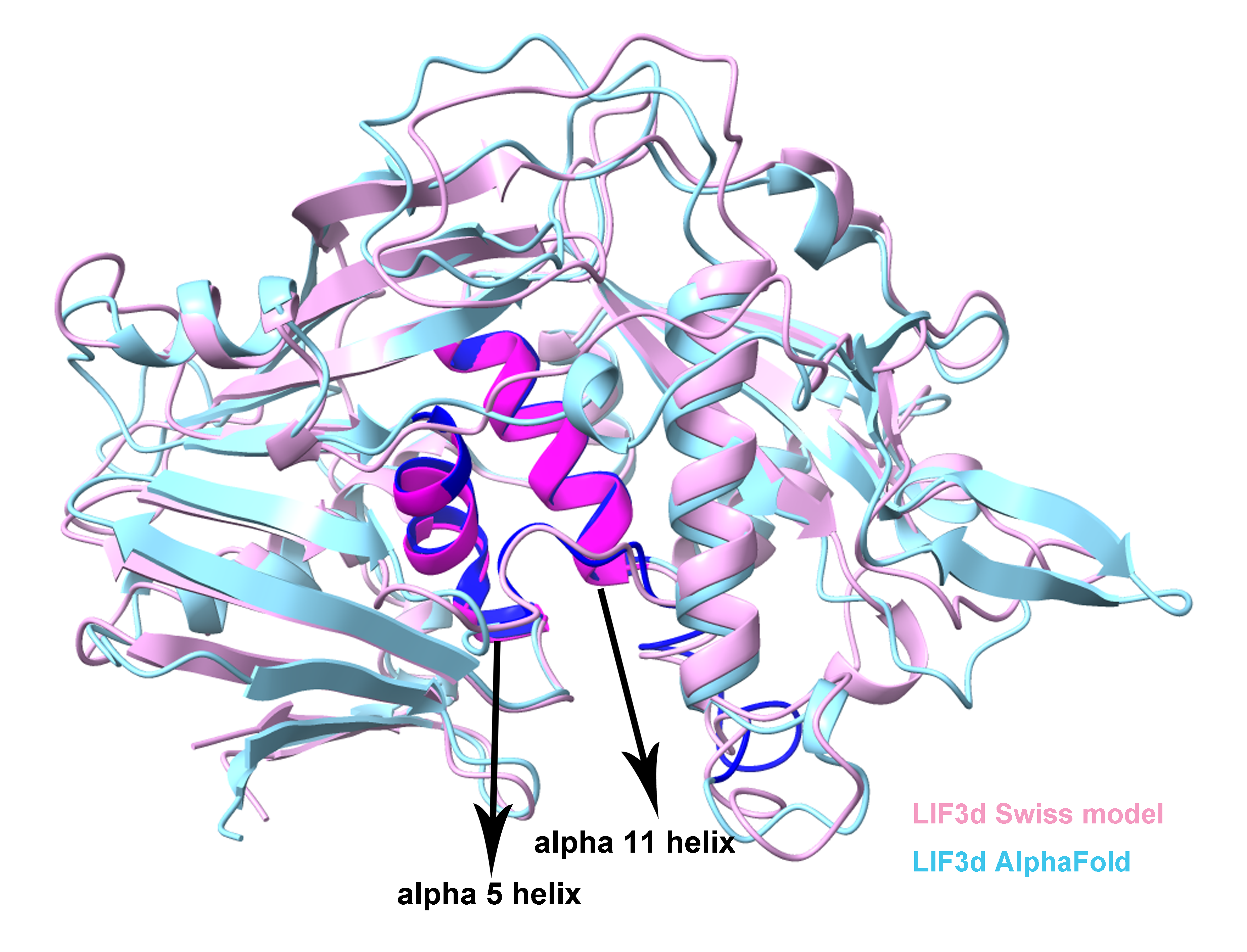

Supplement: Supplementary file 13 [file Image8.TIF]

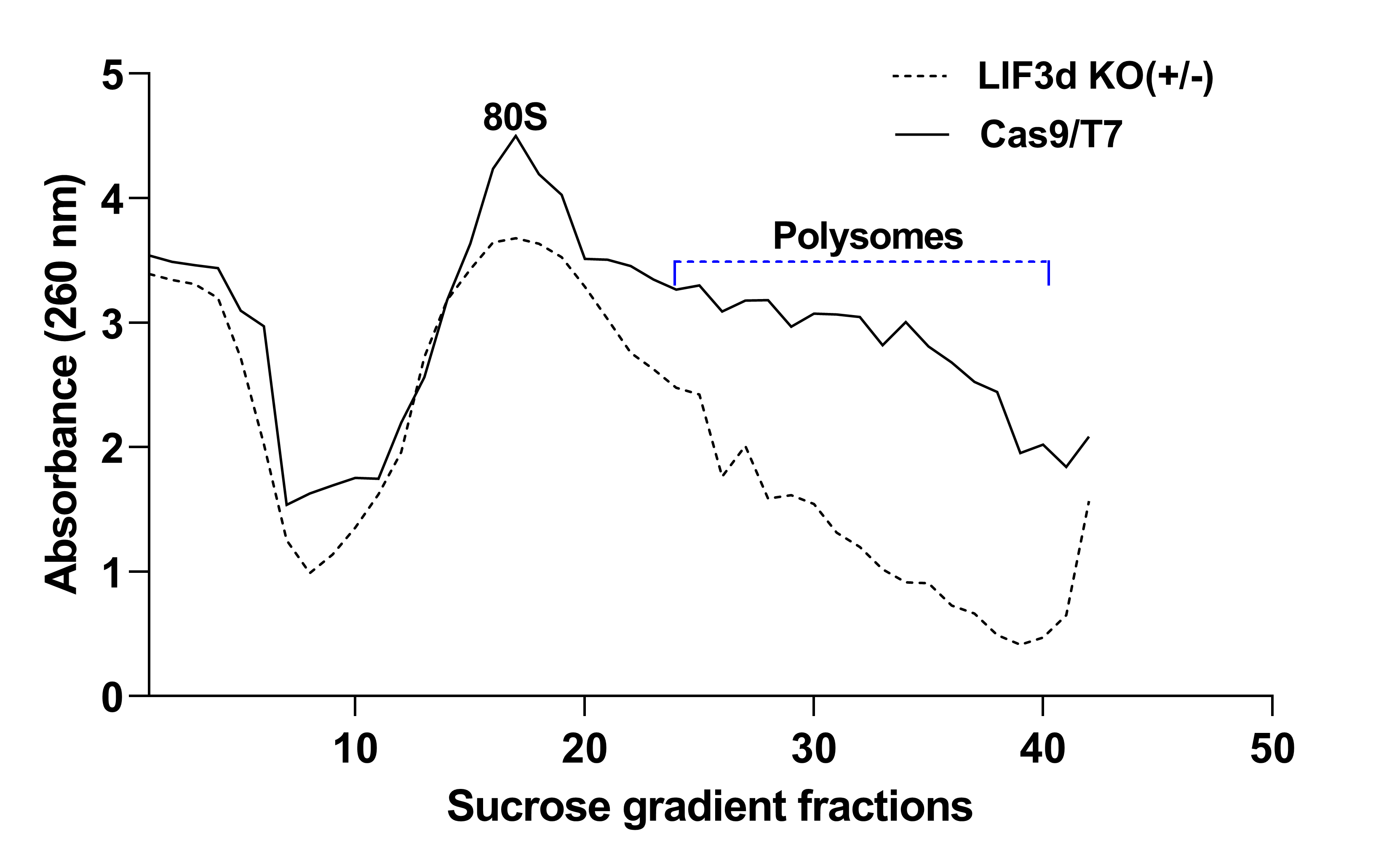

Supplement: Supplementary file 14 [file Image5.TIF]
