# Supplementary material for: LeishIF3d is a non-canonical cap-binding protein in Leishmania
Source: Front Mol Biosci. 2023 May 30;10:1191934. doi: 10.3389/fmolb.2023.1191934 (PMC10266417; doi:10.3389/fmolb.2023.1191934)
Supplement: Supplementary file 4 [file Presentation1.PPTX]

## Slide 1
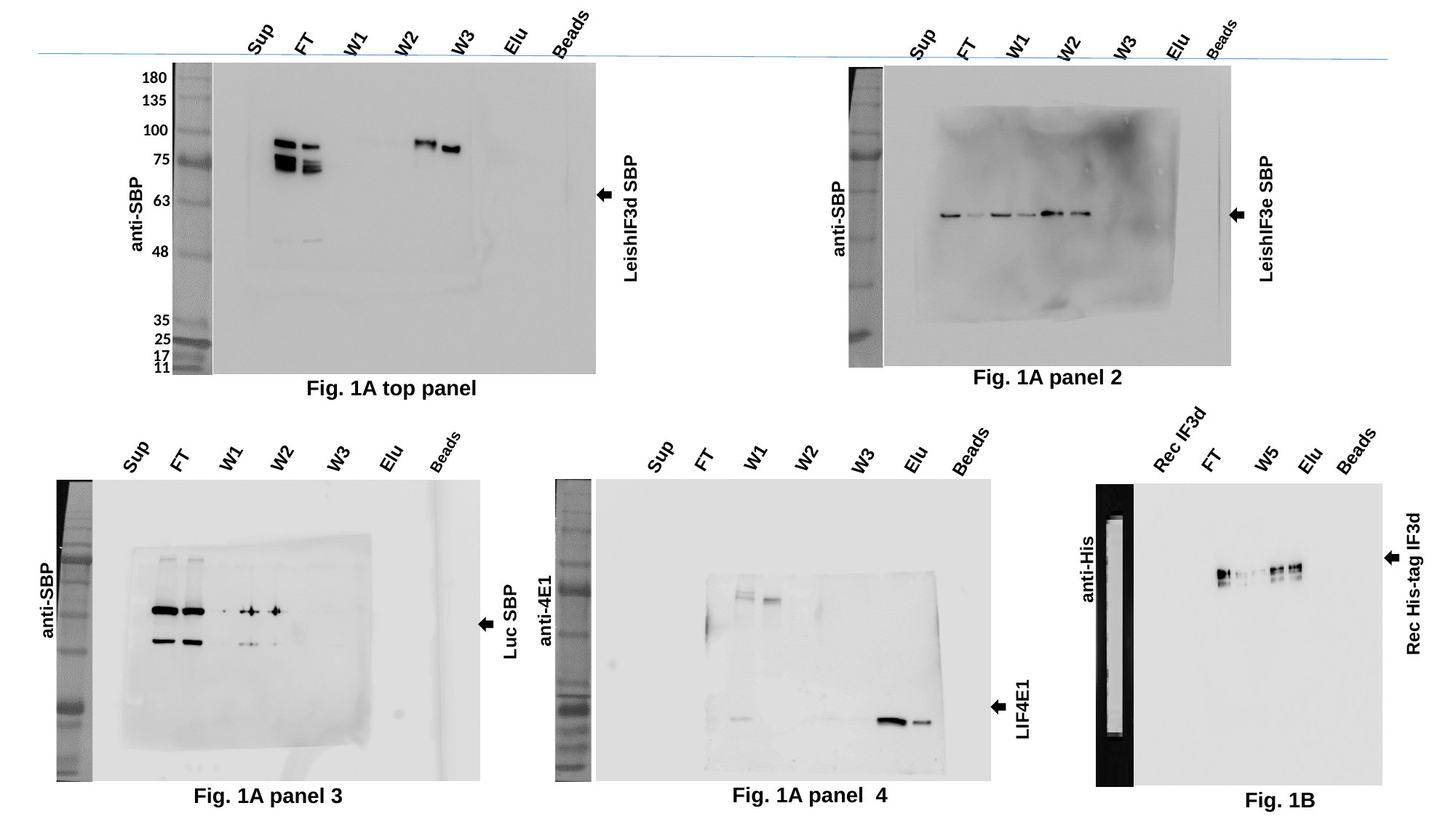

Beads
W3
Beads
W1
W3
W2
W1
Sup
W2
FT
Sup
FT
Elu
Elu
180
135
100
75
anti-SBP
anti-SBP
LeishIF3e SBP
LeishIF3d SBP
63
48
35
25
17
11
Fig. 1A panel 2
Fig. 1A top panel
Beads
Beads
W3
W1
W3
W1
W2
W2
Sup
Sup
FT
FT
Rec IF3d
Beads
Elu
Elu
W5
Elu
FT
anti-His
Rec His-tag IF3d
anti-SBP
anti-4E1
Luc SBP
LIF4E1
Fig. 1A panel 4
Fig. 1A panel 3
Fig. 1B

## Slide 2
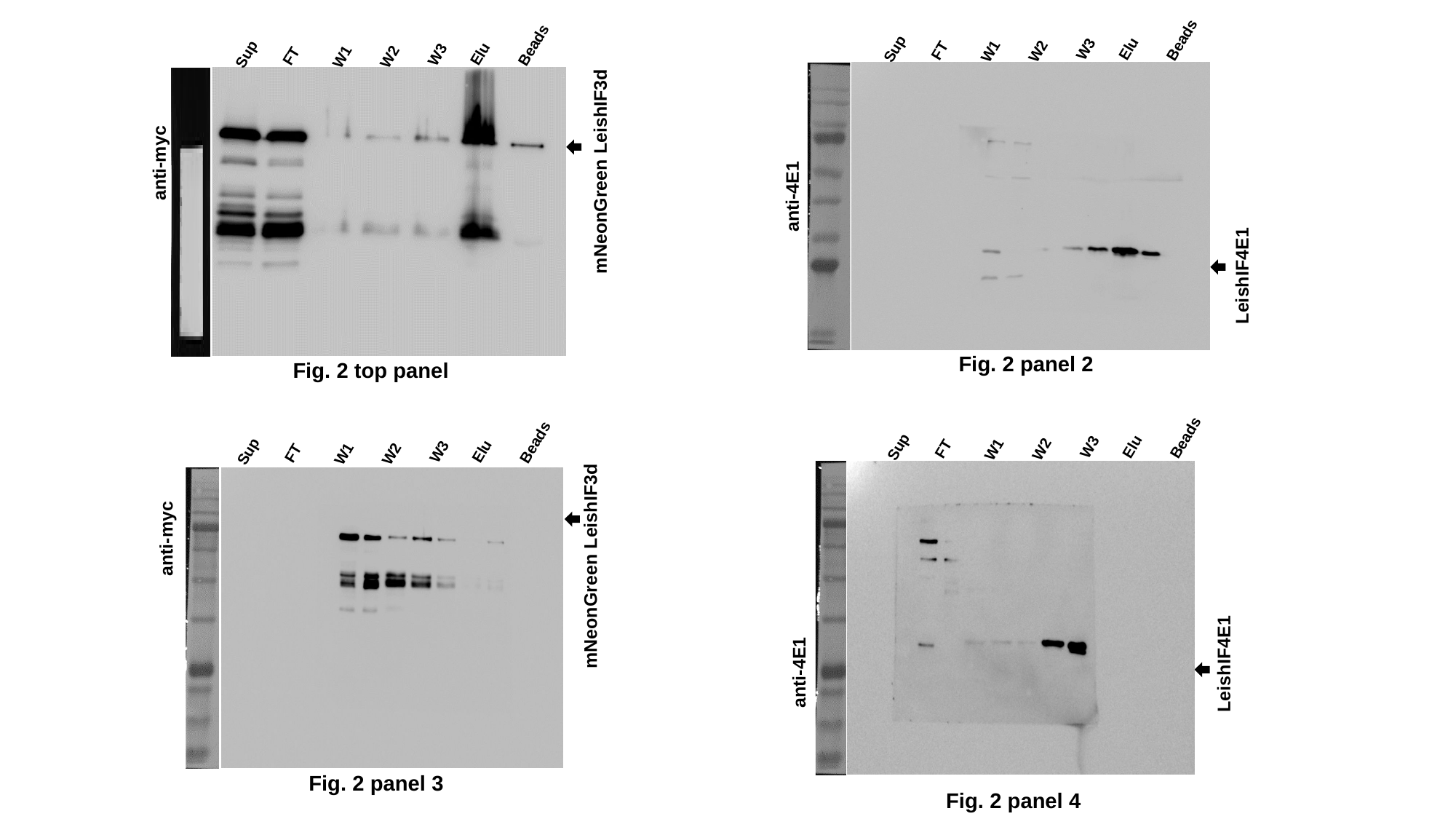

Beads
W3
W1
W2
Sup
FT
Elu
anti-4E1
LeishIF4E1
Fig. 2 panel 2
Beads
W3
W1
W2
Sup
FT
Elu
anti-myc
mNeonGreen LeishIF3d
Beads
W3
W1
W2
Sup
FT
Elu
anti-myc
mNeonGreen LeishIF3d
Fig. 2 panel 3
Fig. 2 top panel
Beads
W3
W1
W2
Sup
FT
Elu
LeishIF4E1
anti-4E1
Fig. 2 panel 4

## Slide 3
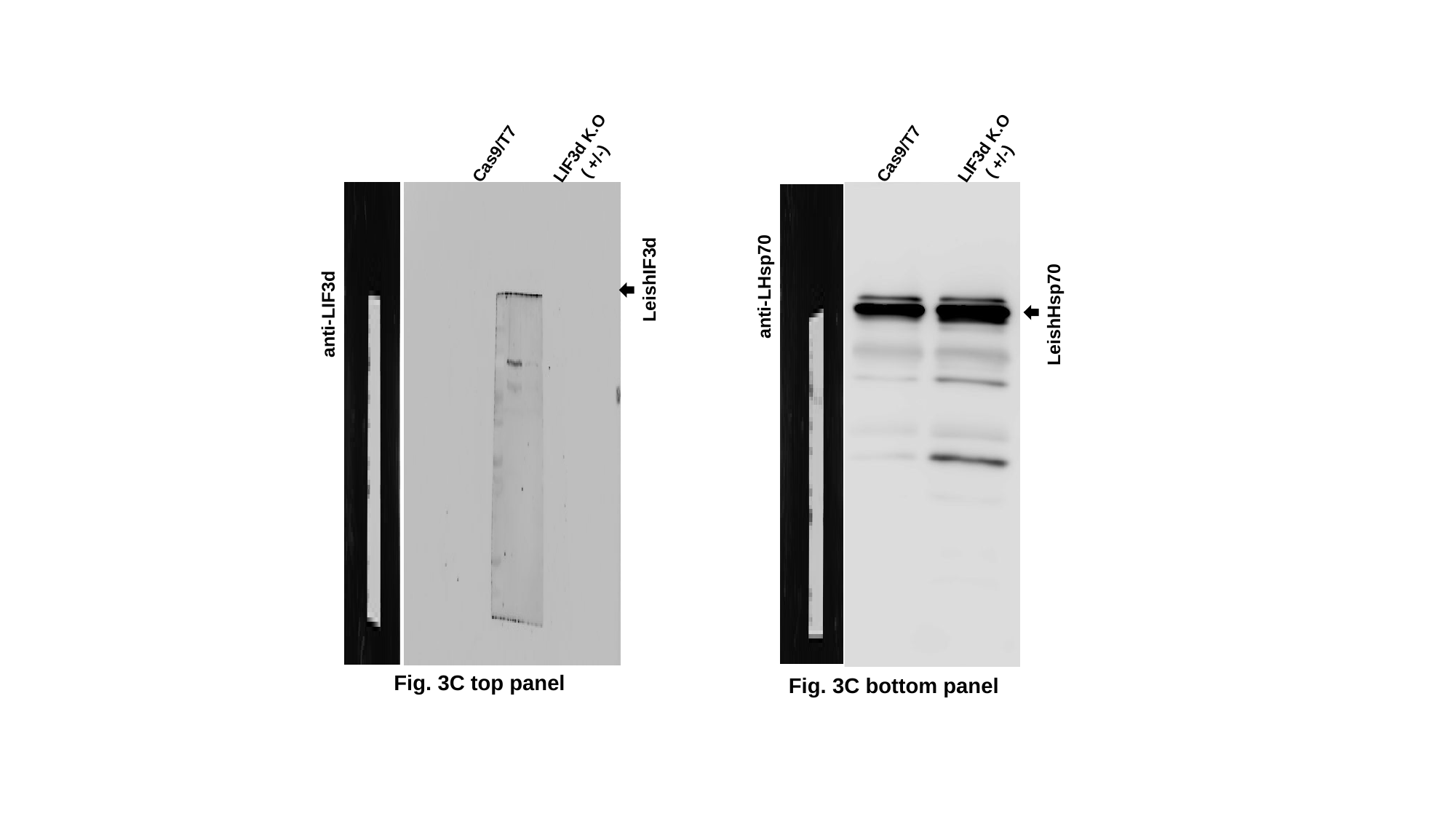

Cas9/T7
 LIF3d K.O
( +/-)
LeishIF3d
anti-LIF3d
 LIF3d K.O
( +/-)
anti-LHsp70
LeishHsp70
Fig. 3C bottom panel
Cas9/T7
Fig. 3C top panel

## Slide 4
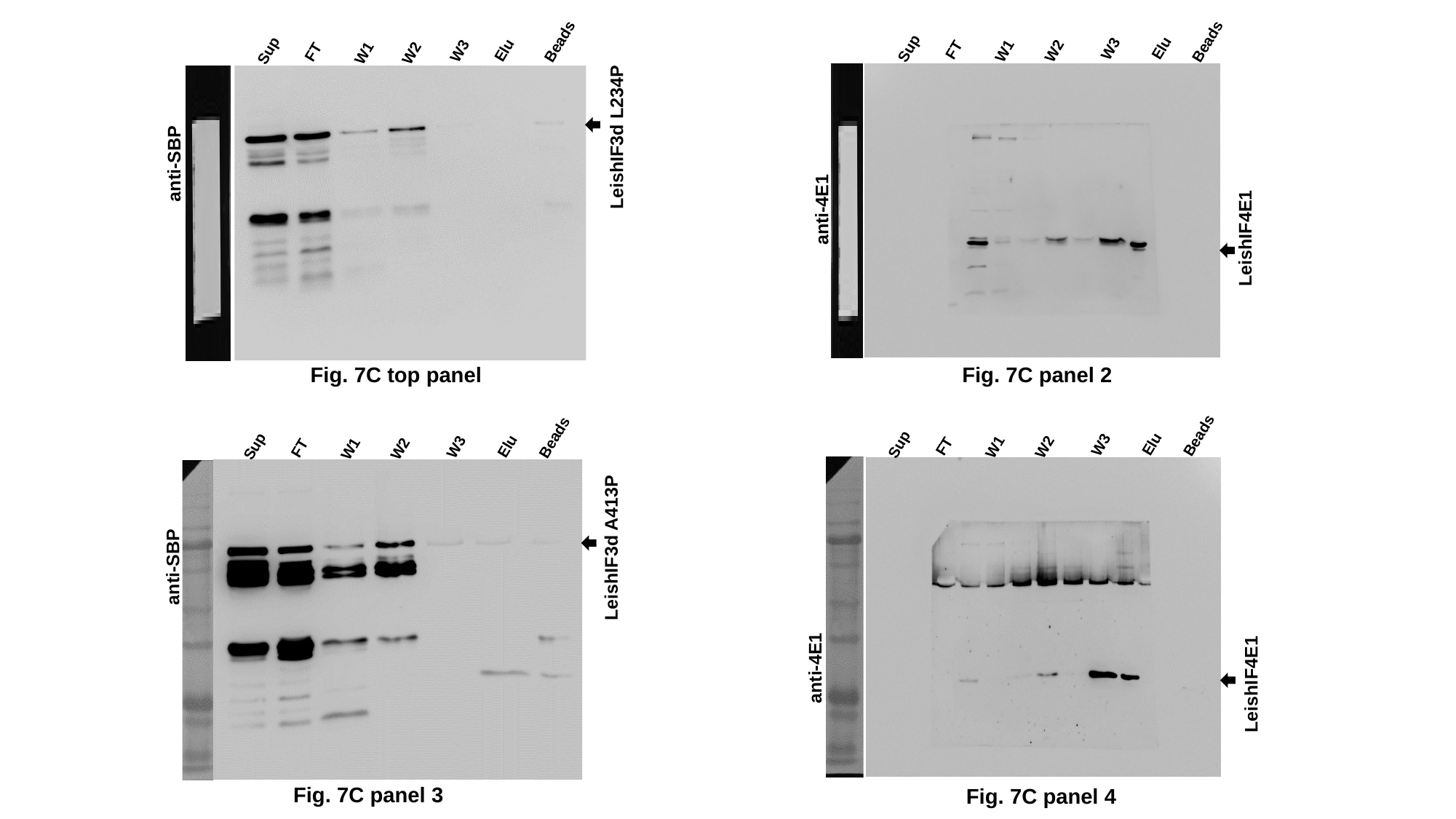

W3
W1
W2
Sup
FT
Elu
anti-4E1
LeishIF4E1
Fig. 7C panel 2
Beads
W3
W1
W2
Sup
FT
Elu
LeishIF3d L234P
anti-SBP
Fig. 7C top panel
Beads
Beads
W3
W1
W2
Sup
FT
Elu
anti-4E1
LeishIF4E1
Fig. 7C panel 4
Beads
W3
W1
W2
Sup
FT
Elu
LeishIF3d A413P
anti-SBP
Fig. 7C panel 3

## Slide 5
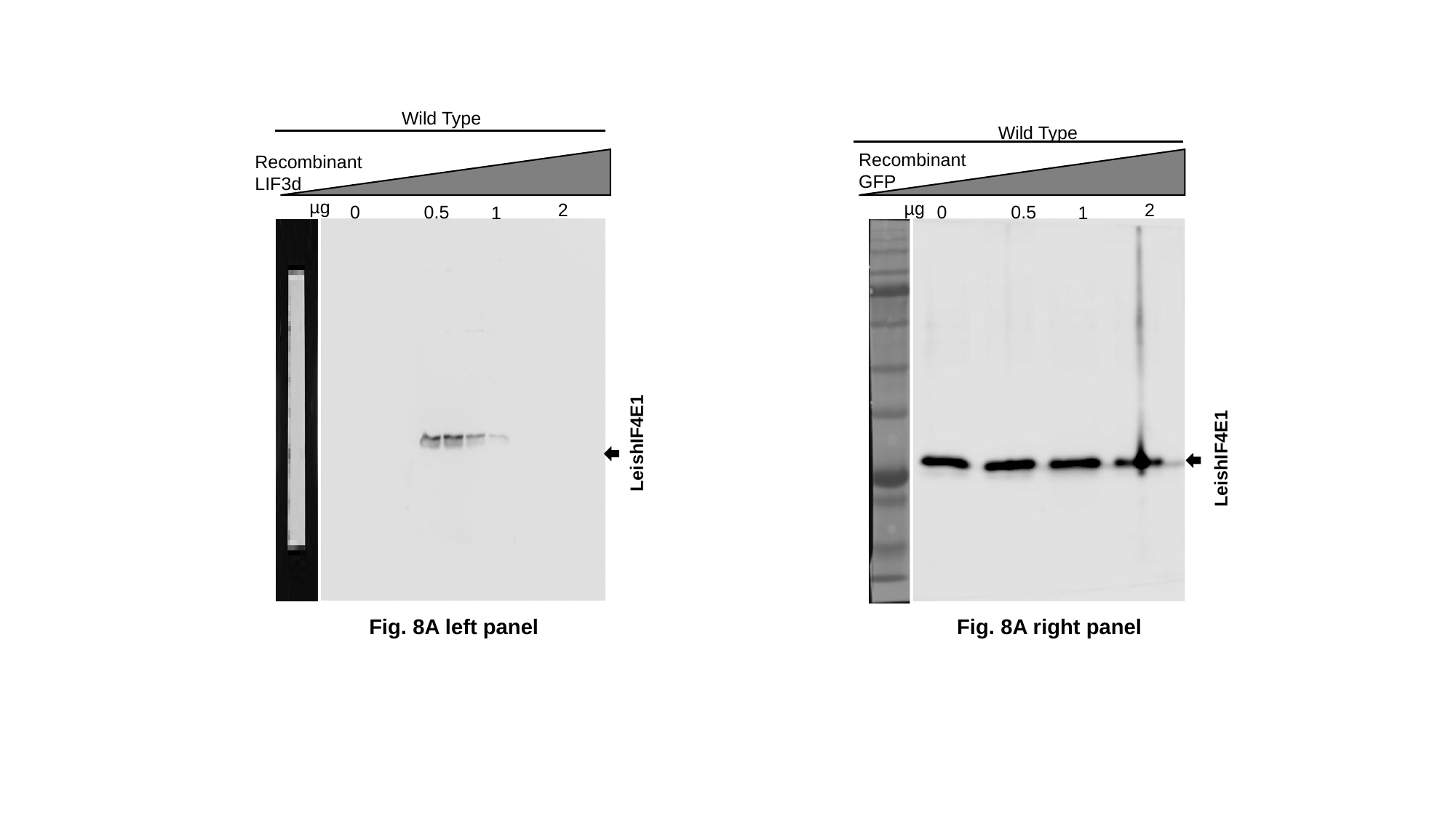

Wild Type
Recombinant
LIF3d
µg
2
0
0.5
1
LeishIF4E1
Fig. 8A left panel
Wild Type
Recombinant
GFP
µg
2
0
0.5
1
LeishIF4E1
Fig. 8A right panel

## Slide 6
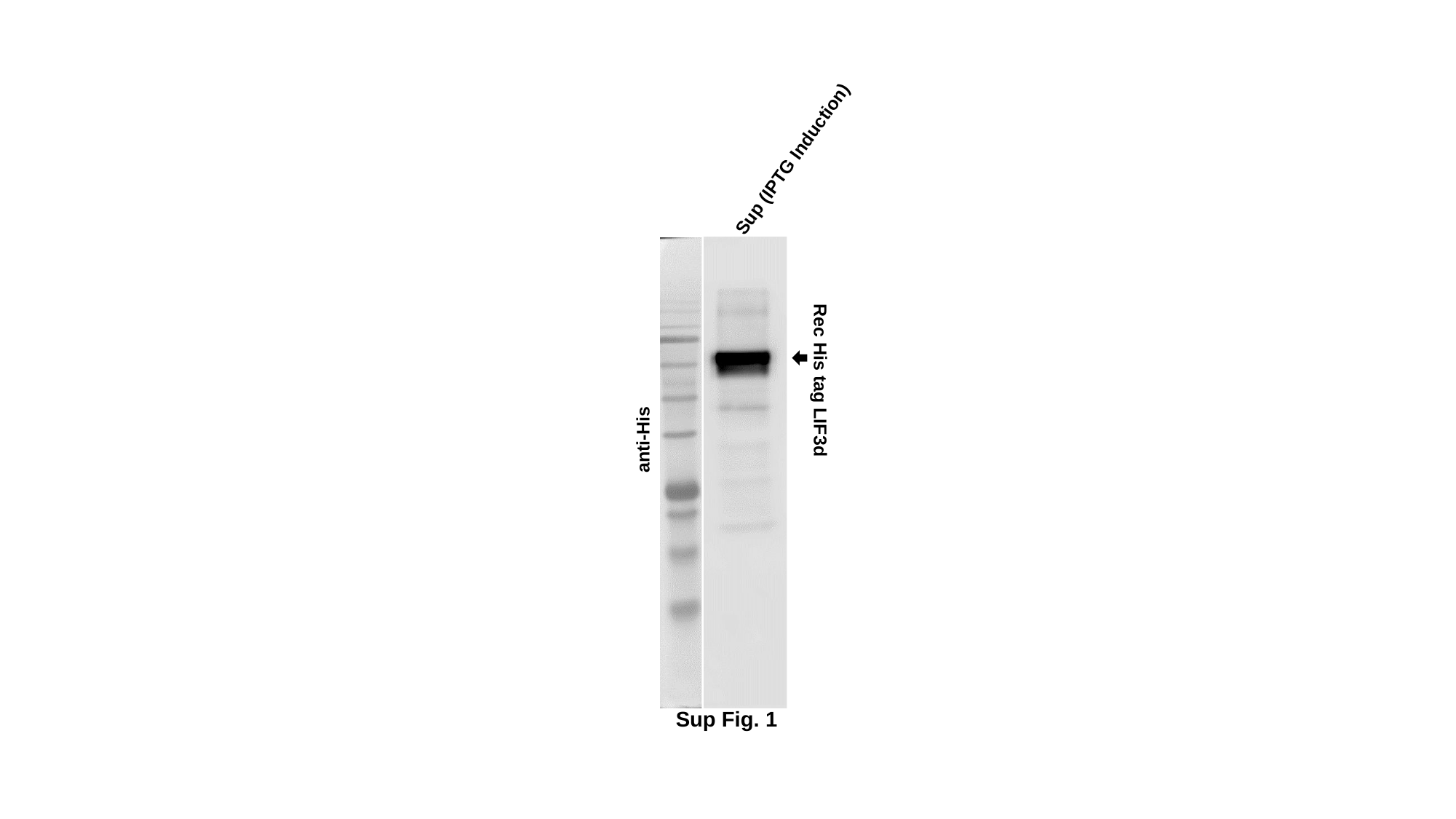

Sup (IPTG Induction)
Rec His tag LIF3d
anti-His
Sup Fig. 1
