# Supplementary material for: LeishIF3d is a non-canonical cap-binding protein in Leishmania
Source: Front Mol Biosci. 2023 May 30;10:1191934. doi: 10.3389/fmolb.2023.1191934 (PMC10266417; doi:10.3389/fmolb.2023.1191934)
Supplement: Supplementary file 10 [file DataSheet1.PDF]

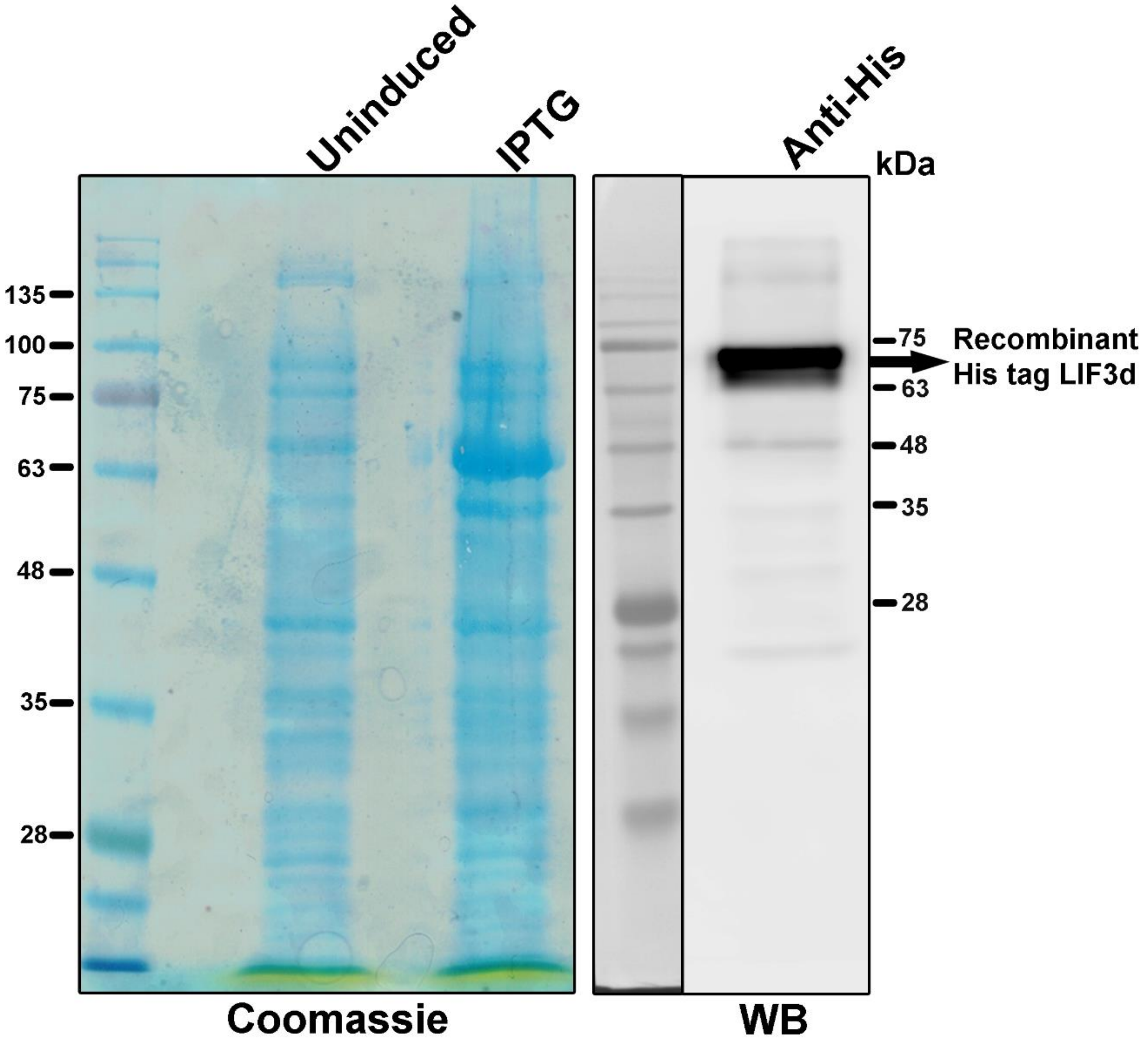

Figure S1. Affinity purification of His tag LeishIF3d. The open reading frame (1596bp) of LeishIF3d was cloned into the pHIS-parallel vector, fused with a HIS tag, and expressed in BL-21 cells. Left panel: represents the induction of expressed LeishIF3d in BL-21 cells using 1mM IPTG (Coomassie). Right panel: Expression of LeishIF3d was further confirmed by the specific antibodies against the His-tag (WB).

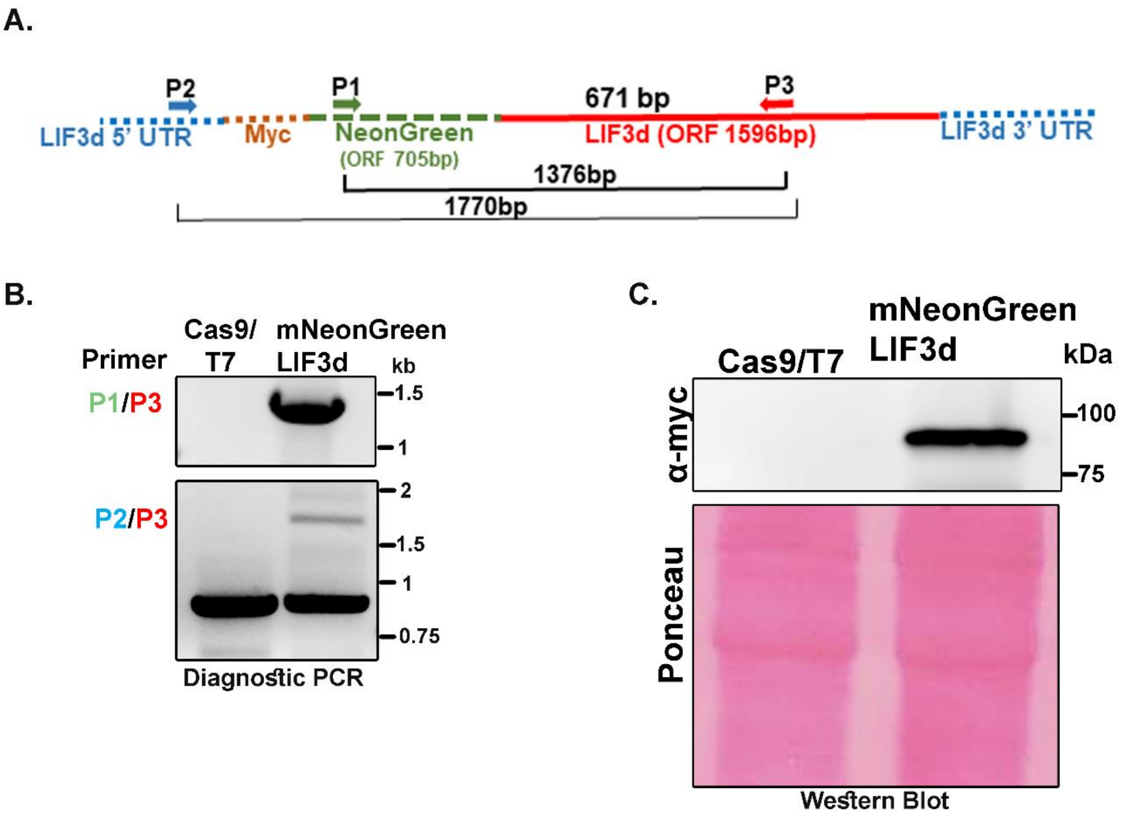

Figure S2. CRISPR-Cas9 mediated mNeonGreen tagging of LeishIF3d. (A) Schematic representation of the mNeonGreen tag fused to the endogenous LeishIF3d. The primers (arrows) used to diagnose the endogenous tagging of LeishIF3d are marked with arrows. The primers derived from the LeishIF3d UTR and ORF are shown in blue and red, respectively. The primer derived from the mNeoGreen gene is shown in green. **(B)** A diagnostic PCR was carried out to confirm the endogenous tagging from the genome of *L. mexicana*. Genomic DNA that was extracted from the mNeonGreen tagged LeishIF3d cells and from the control cell line of *L. mexicana* expressing Cas9/T7 was used as a template for PCR using primers derived from the LeishIF3d ORF and from the G418 resistance gene ORF.

(C) Western analysis identifying the endogenously mNeonGreen tag LeishIF3d cells, and not in the Cas9/T7 control cells was performed using monoclonal anti-myc antibodies. Ponceau staining of the blot served as a loading control.

A.

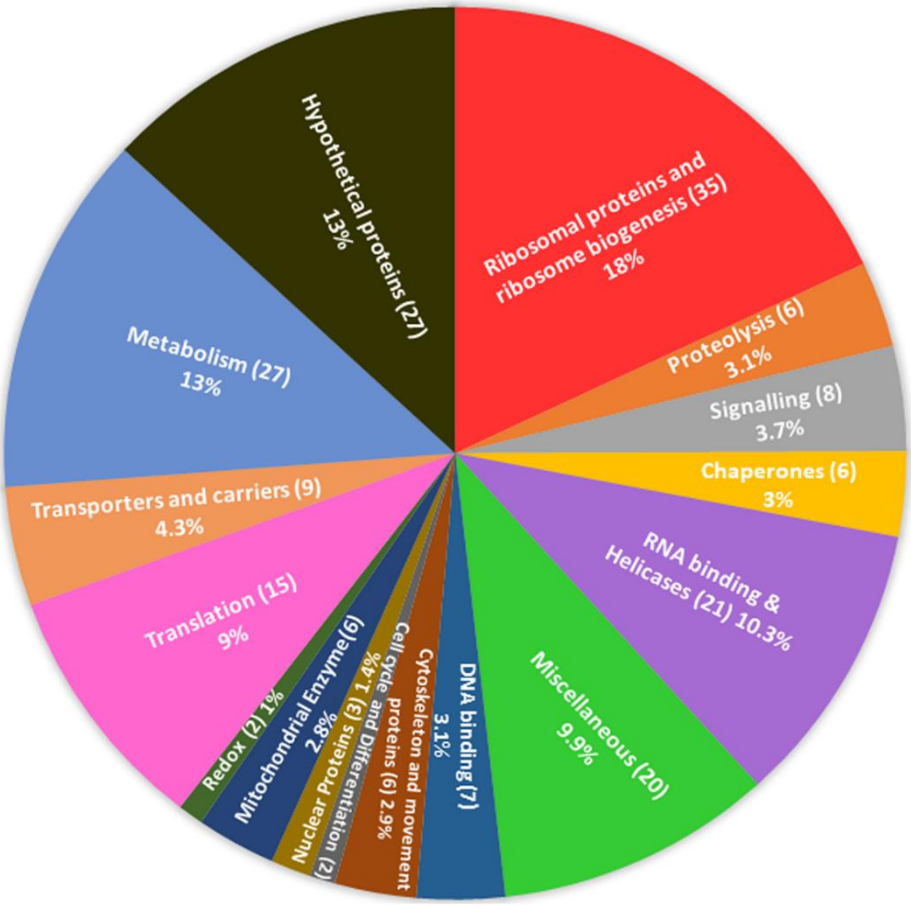

B.

List of Translation factor subunits
Eukaryotic translation initiation factor 3 subunit “a”, “b”, “c”, “e” “f”, “h”, “i”, “l”, “k”
Eukaryotic translation initiation factor 1
Eukaryotic translation initiation factor 2 subunit, Translation initiation factor eIF-2B subunit epsilon
Eukaryotic translation initiation factor 5A
Elongation factor-1 beta, elongation factor-1 gamma

Figure S3. Categorized pulled-down proteomic content over mNeonGreen tagged LeishIF3d. (A) The proteomic content of endogenously mNeonGreen tagged LeishIF4E3 immuno-precipitated over anti-c-Myc agarose beads was determined by LC-MS/MS analysis and compared to a control pull down performed with control promastigotes expressing the episomal mNeonGreen tag. All analyses were performed in triplicates and in the same run. The proteins were identified by the MaxQuant software using TriTrypDB database annotations.

Differences between the proteomic contents of the LeishIF3d and control pulled-down fractions were determined using the Perseus statistical tool. Proteins enriched by two fold with a $p<0.05$ were categorized according to their function. % represents the accumulated intensities in each category. The number of proteins in each category is also marked. **(B)** LC-MS/MS analysis of the eIF3d pull-down complex shows subunits derived from co-purification with proteins annotated as eIF1, eIF2, eIF5 and the eIF3 complexes. The eIF3d interactome also contains elongation factors.

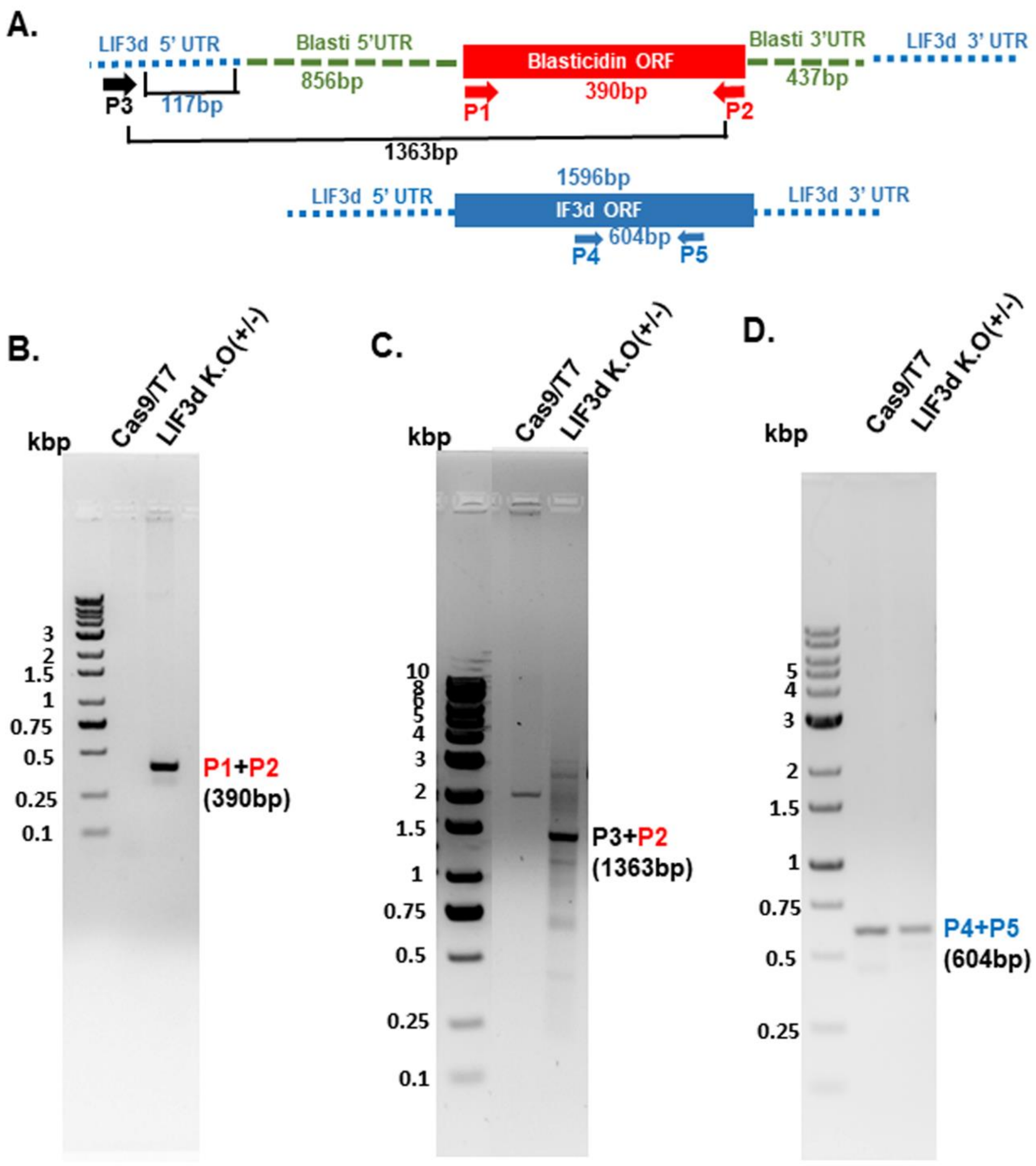

Figure S4. **CRISPR-Cas9 mediated hemizygous deletion of LeishIF3d(+/-)**

Diagnostic PCR was performed to confirm the deletion of single allele of LeishIF3d. Genomic DNA of *L. mexicana* was extracted from the LeishIF3d(+/-) mutant and from the *L. mexicana* Cas9/T7 cells. PCR was performed using primers derived from the ORF of blasticidin resistance gene (A), LeishIF3d 5' UTR, and blasticidin reverse (B), and the ORF of LeishIF3d resistance gene (C).

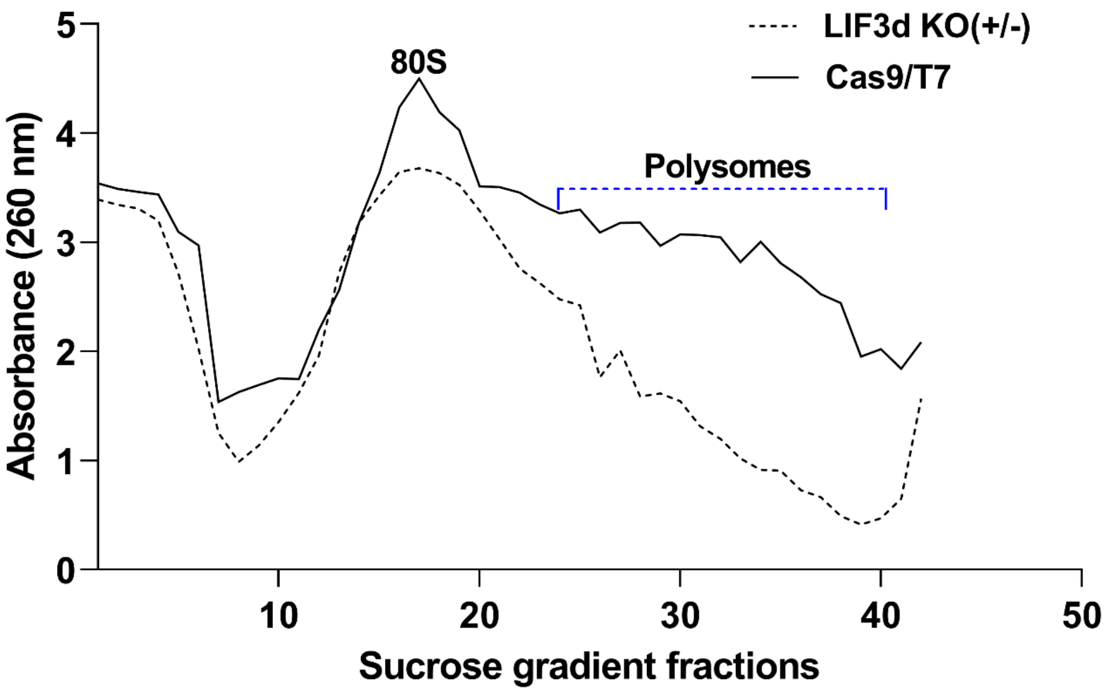

Figure S5. **Polysome distribution in cell extracts derived from LeishIF3d(+/-) and Cas9/T7 cells**. Cells were lysed, the extracts were clarified by centrifugation and loaded on 10 to 50% sucrose gradients that were centrifuged at 35,000 rpm during 160min at 4°C, in a SW40 rotor. Fractions were collected from the top, and the optical density at 260 nm was measured.

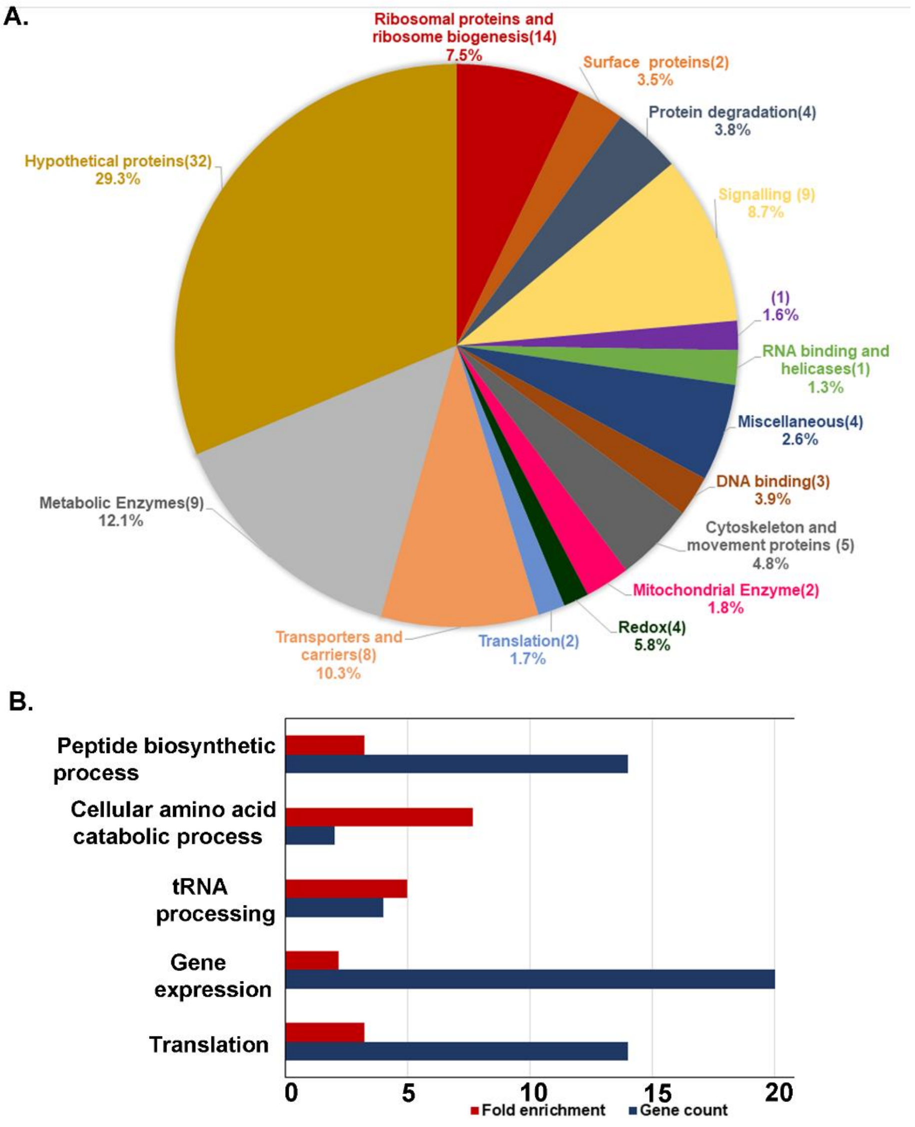

Figure S6. **The categorized proteome of the downregulated proteins in LeishIF3d(+/-) mutant cells as compared to Cas9/T7 control cells.** The proteomic content of LeishIF3d(+/-) and Cas9/T7 cells was determined by LC-MS/MS analysis, in triplicates. Raw MS data were analyzed and quantified using the MaxQuant software and the peptide data were searched against the annotated L. mexicana proteins listed in TriTrypDB. The summed intensities of the peptides that served to identify the individual proteins were used to quantify changes in the proteomic content of specific proteins. Statistical analysis was done using the Perseus software. Proteins that were down regulated in the LeishIF3d mutant by at least log2 of 2-fold as

compared to Cas9/T7 cell extracts, with $p<0.05$ are shown. (A) Proteins in LeishIF3d(+/-) that were downregulated (>1 log2 fold) as compared to Cas9/T7 extracts were clustered manually into functional categories. The pie chart represents the summed intensities of upregulated proteins in each category in the LeishIF3d(+/-) mutant. Numbers in brackets indicate the number of proteins in each category, and % represents their summed relative intensity in the analysis. (B) Enriched proteins were classified by the GO enrichment tool in TriTrypDB, based on the Biological process. The threshold for the calculated enrichment of proteins based on their GO terms was set as log2 of 1-fold, with $q<0.05$. This threshold eliminated most of the general groups that represented parental GO terms. GO terms for which only a single protein was annotated were filtered out as well. In some cases, GO terms that were included in other functional terms are not shown, leaving only the representative GO term.

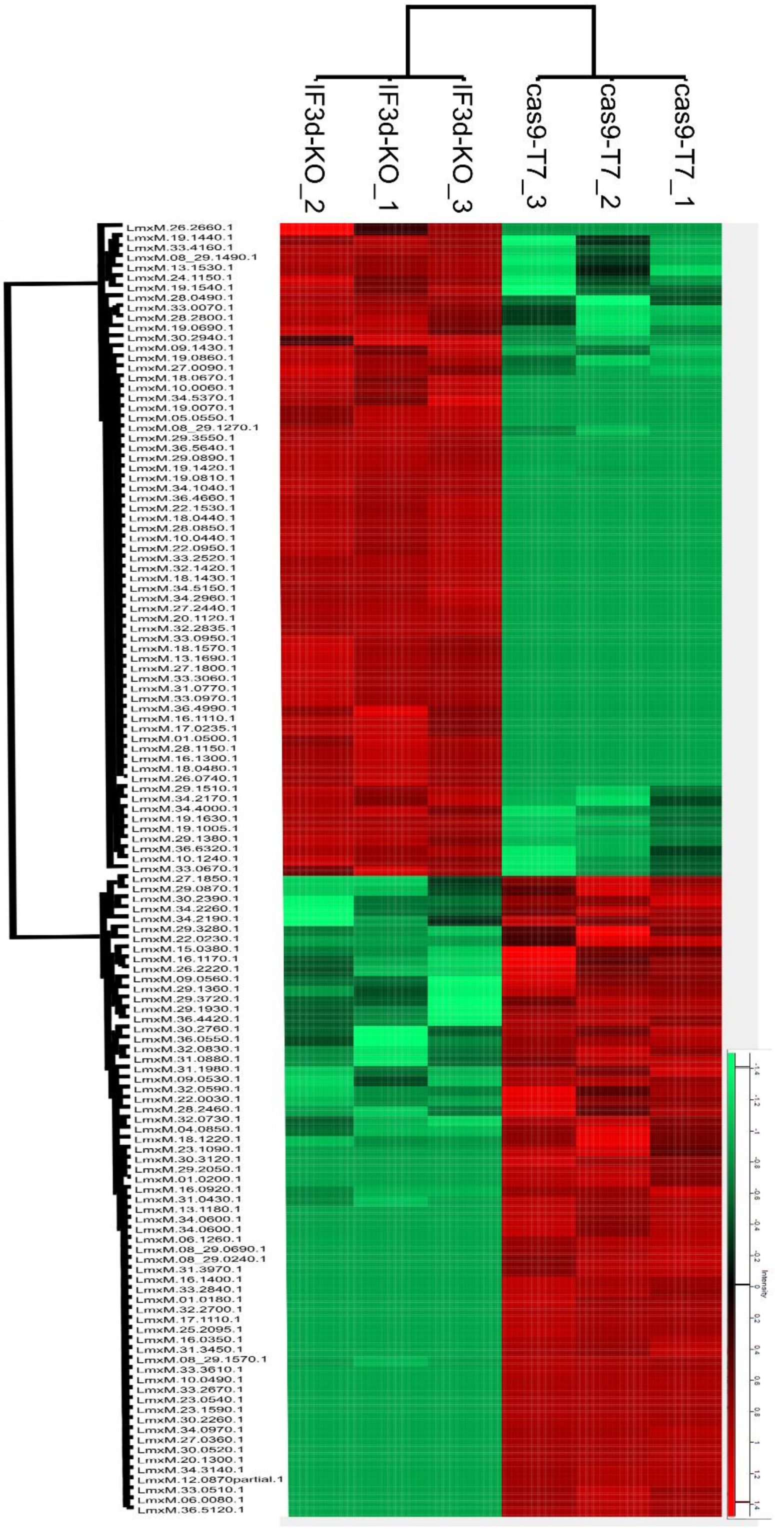

Figure S7. **The hemizygous deletion of LeishIF3d alters the proteomic profile of the LeishIF3d(+/-) cells.** The proteomic content of LeishIF3d(+/-) and Cas9/T7 cells was determined in triplicates by LC-MS/MS. The heatmap displays the intensities of differentially

expressed proteins in the LeishIF3d mutant by at least log2 = 1 as compared to Cas9/T7 cell extracts, $q<0.05$ with Hierarchical clustering (using Perseus software platform). Protein intensities were normalized by Z-score. The color scale illustrates the relative expression level of each protein across the 3 repeat samples; red and green indicate higher and lower expression, respectively. The different columns show the three biological repeats of each sample; the rows represent the individual proteins.

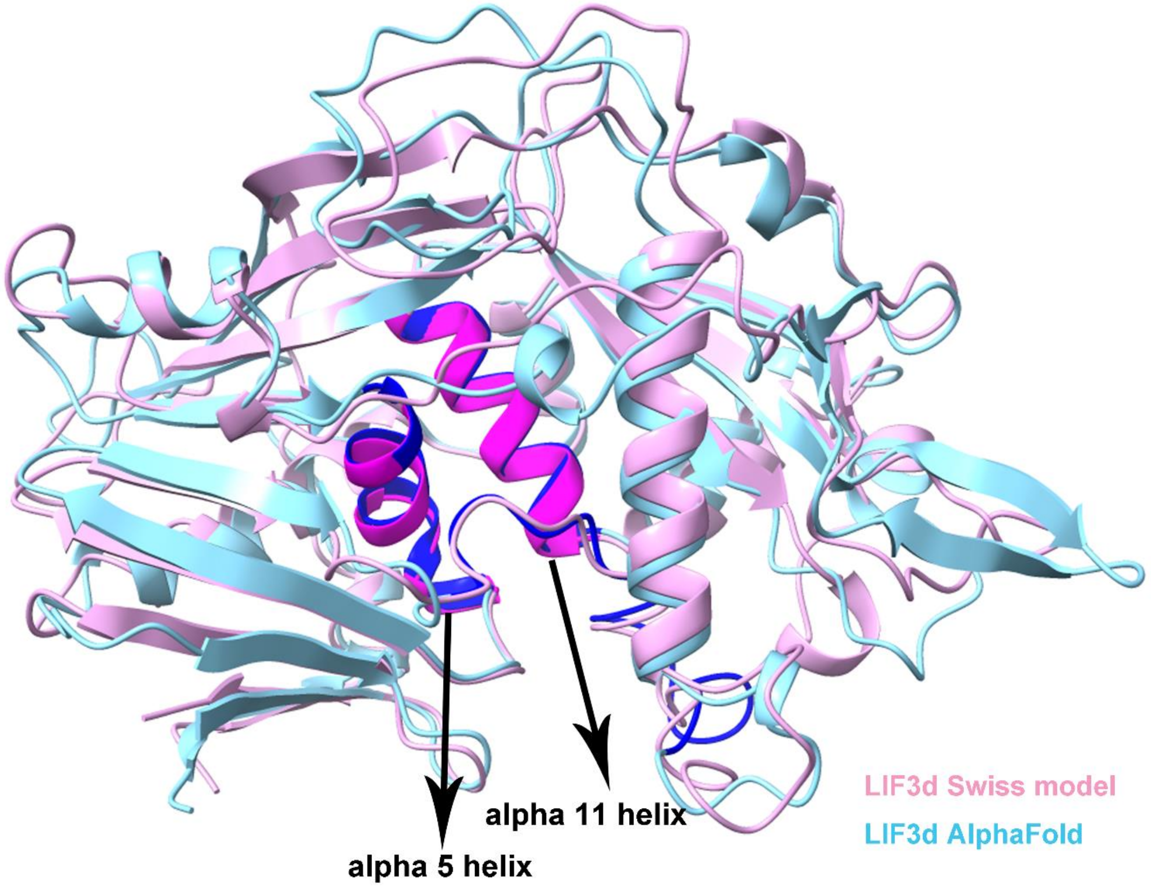

Figure S8. ***L. mexicana* IF3d structural homology modeling.** The structural homology of *Leishmania mexicana* IF3d (143-505 aa; LmxM.29.3040) was modeled via the AlphaFold server (pink). Another model of *L. mexicana* IF3d was generated using the SWISS-MODEL (blue) server based on the *Nasonia vitripennis* eIF3d solved structure (5k4b) to validate the alpha fold model. The *L. mexicana* IF3d models that were generated using AlphaFold and SWISS-MODEL were also superposed using ChimeraX.
